# Supplementary material for: A High-Quality Grapevine Downy Mildew Genome Assembly Reveals Rapidly Evolving and Lineage-Specific Putative Host Adaptation Genes
Source: Genome Biol Evol. 2019 Mar 7;11(3):954–69. doi: 10.1093/gbe/evz048 (PMC6660063; doi:10.1093/gbe/evz048)
Supplement: Supplementary Data [file evz048_supp.pdf]

SUPPLEMENTARY MATERIALS FOR "A high-quality grapevine downy mildew genome assembly reveals rapidly evolving and lineage-specific putative host adaptation genes"

Yann Dussert[1]*, Isabelle D. Mazet[1], Carole Couture[1], Jérôme Gouzy[2], Marie-Christine Piron[3], Claire Kuchly[4], Olivier Bouchez[4], Claude Rispe[5], Pere Mestre[3], François Delmotte[1]

[1]SAVE, INRA, Bordeaux Sciences Agro, Villenave d'Ornon, France; [2]LIPM, INRA, Université de Toulouse, CNRS, Castanet-Tolosan, France; [3]SVQV, INRA, Université de Strasbourg, Colmar, France; [4]US 1426 GeT-PlaGe, Genotoul, INRA, Castanet-Tolosan, France; [5]BIOEPAR, INRA, ONIRIS, Université Bretagne Loire, Nantes, France

*Author for correspondence: Yann Dussert, SAVE, INRA, Bordeaux Sciences Agro, Villenave d'Ornon, France, dussert.yann@gmail.com

This document includes:

Supplementary note

Supplementary figure 1 Comparison between the long-read and short-read assemblies of the *Pl. viticola* INRA-PV221 isolate.

Supplementary figure 2 K-mer comparison between the corrected PacBio reads and the *Pl. viticola* genome assembly.

Supplementary figure 3 Synteny between *Pl. viticola* and *Pl. muralis*, *Pl. halstedii*, *Ph. infestans* and *Py. ultimum*.

Supplementary figure 4 Phylogenetic relationships between oomycete species.

Supplementary figure 5 Sequence logo of *Pl. viticola* and *Pl. muralis* proteins with a WY motif but no RxLR signal.

Supplementary figure 6 Local gene density and organization in *Pl. muralis.*

Supplementary figure 7 Distribution of distances to the closest gene and repeat sequence in (A) *Pl. viticola* and (B) *Pl. muralis* for different gene categories.

Supplementary table 1 Oomycete species and data origin.

Supplementary table 2 Assembly statistics for published *Pl. viticola* genome sequences.

Supplementary table 3 Type of repeat sequences in the *Pl. viticola* and *Pl. muralis* genomes.

Supplementary table 4 Statistics of the synteny analysis between the genomes of *Pl. viticola* and four other oomycete species.

Supplementary table 5 GO enrichment analysis for ortholog groups missing in oomycete biotrophic species.

Supplementary table 6 Number of flavoproteins in biotrophic and non-biotrophic oomycetes.

Supplementary table 7 KEGG annotation of *Ph. infestans* orthologs missing in biotrophic species.

Supplementary table 8 GO enrichment analysis for the secretome of *Pl. viticola.*

Supplementary table 9 GO enrichment analysis for the secretome of *Pl. muralis.*

Supplementary table 10 Results of the detection of candidate RxLR effector genes in *Pl. viticola* and *Pl. muralis.*

Supplementary table 11 Orientation and intergenic distances for adjacent gene pairs *in Pl. viticola* and *Pl. muralis*.

Supplementary table 12 *Pl. viticola* and *Pl. muralis* ortholog pairs with a dN/dS above 1.

Supplementary table 13 *Pl. viticola* genes with a significant signal in the branch-site test.

Supplementary table 14 *Pl. muralis* genes with a significant signal in the branch-site test.

SUPPLEMENTARY NOTE

Genome assembly for *Pl. viticola*

The PBcR wgs8.3rc2 assembly pipeline (Berlin *et al.* 2015) was used to perform read correction and assemble the corrected reads with the following parameters:
i) only raw reads longer than 5 kb were considered
ii) 50x of corrected reads were collected (assembleCoverage=50)
iii) the unitigger bogart was tuned to handle heterozygosity (utgErrorRate=0.05 and utgMergeErrorRate=0.05).
Corrected reads were also assembled using FALCON (Chin *et al.* 2016) with the following parameters:
fc_ovlp_filter: --max_diff 240 --max_cov 150 --min_cov 30 --bestn 50 --min_len 5000
fc_ovlp_to_graph: --min_len 5000

As indicate in the main text of the paper, while the PBcR assembly was around twice the expected size of the genome due to high heterozygosity, it was more comprehensive than the FALCON assembly, so we removed redundancy in the PBcR assembly using an ad-hoc procedure: the contigs representing the alternative haplotype, defined as contigs fully included in larger contigs (with structural variations allowed), were removed. In a first step, an all vs all comparison was performed (megablast, min score=1000, minimum identity percentage=90), then syntenic HSPs were chained using LYNX (available in the *Pl. viticola* genome data repository at doi.org/10.15454/4NYHD6). A contig was removed when it was the shorter one of the pair and the chain spanned more than 70% of its length.
As some contigs were bacterial contaminants (mainly *Pseudomonas extremorientalis* and a *Stenotrophomonas*), FALCON was used to assemble bacterial genomes using parameters tuned for low coverage assembly (fc_ovlp_filter: --max_diff 50 --max_cov 50 --min_cov 1 --bestn 10 --min_len 5000, fc_ovlp_to_graph: --min_len 5000). The assembled bacterial genomes were used to remove contaminant-related contigs in the PBcR assembly (NCBI-TBLASTX, -e-value $10e^{-20}$, GC content > 56%).
Finally the PBcR contigs were scaffolded (19 gaps spanned) using a FALCON assembly (fc_ovlp_filter: --max_diff 10000 --max_cov 300 --min_cov 3 --bestn 1000 --min_len 5000).
Illumina paired-end reads (SRA accession numbers: SRX1970160, SRX1970161, SRX1970162) and 3 kb mate-pair reads (SRX1970164) used for a previous draft genome of the same isolate (Dussert *et al.* 2016) were mapped on the assembly sequence with BWA (Li & Durbin 2009). After running Pilon (parameters: --diploid --vcf --changes --fix bases) a first time, quality of the genome sequence measured with BUSCO v2 (Simão *et al.* 2015) with the Alveolata-Stramenopiles dataset was low (85% of expected genes). After observation of the assembly sequence and of the read mapping in a genome browser, we concluded that intra-individual variations for indel polymorphism were not properly phased (i.e. indels close to each other and belonging to the two different haplotypes were often in the consensus assembly sequence) and were causing frame shifts in coding sequences. This was not corrected by Pilon, since the software only considers one site at

a time. Heterozygous nucleotide sites with an error in the assembly sequence (for example, sites that were A/T in the mapping data but C or G in the assembly) were also not corrected. To resolve these issues, the VCF output of Pilon was parsed using a custom script (available in the *Pl. viticola* genome data repository) to correct heterozygous nucleotide sites with an error and find pairs of neighboring heterozygous indels (distance < 10 bp) and correct only one of them. This procedure was performed three times, and for the final correction round, proposed corrections were checked on a genome browser and accepted or not.

Genome assembly for *Pl. muralis*

Since *Pl. muralis* sporangia and sporiangiophores were collected in non-sterile conditions outside the lab, contaminants were expected. All Illumina reads from *Pl. muralis* were first assembled with SPAdes 3.9.0 (Bankevich *et al.* 2012) (k-mer sizes: 21, 33, 55, 77). Contigs were then aligned against the NCBI nt database with BLASTN+, and reads were re-mapped on the contigs using BWA, keeping only properly paired reads. Reads mapping to contigs identified as contaminants were discarded, and a new assembly was carried out. This procedure was performed until no known contaminants were detected (three times). Since the large majority of contaminant contigs had a very low read coverage, we also discarded contigs with a coverage inferior to 20x and no blast hits with oomycete sequences. It is very likely that some sequences belonging to *Pl. muralis* were discarded with this approach, but it was deemed preferable to reduce contaminants to a minimum. Finally, we only kept contigs with a size superior to 1 kb. The median read coverage for the final assembly was 120x.

Annotation

Gene annotation. For *Pl. viticola*, after aligning RNA-seq reads with STAR 2.4 (Dobin *et al.* 2013) (--alignIntronMax 20000 --outFilterMismatchNmax 8, keeping only properly paired reads and discarding secondary alignments), genes were first predicted on the softmasked genome (de novo repeats and Repbase db) with BRAKER 1.8 (Hoff *et al.* 2016) (--alternatives-from-evidence=false; GeneMark-ES Suite 4.21, AUGUSTUS 3.1). Aligned RNA-seq reads were also assembled into transcripts using Trinity release 20140717 (Grabherr *et al.* 2011) (genome-guided assembly, --jaccard-clip), and the gene predictor SNAP (Korf 2004) was trained with the gene models from BRAKER. SNAP was then run on the softmasked genome in MAKER 2.31 (Holt & Yandell 2011), with the assembled transcripts and the proteomes of 9 other oomycetes (Supplementary table 1) as evidence. Observation of the results from BRAKER and SNAP in a genome browser indicated that gene models from BRAKER were of much higher quality. We chose to keep all BRAKER gene models, and only SNAP gene models not overlapping with BRAKER ones. A final MAKER run was then carried out to predict UTRs sequences, but this information was not used for any analyses, since observation of the annotation in a genome browser indicated that the quality of the predicted UTRs was low, with a high prevalence of too long sequences.

For *Pl. muralis*, for which no RNA data was available, we first made an initial MAKER run with AUGUSTUS trained with the *Pl. viticola* gene annotation as gene predictor and *Pl. viticola* proteins as evidence. AUGUSTUS and SNAP were then trained with this preliminary annotation. As

previously, SNAP gene models were of lower quality, so we only kept SNAP models not overlapping AUGUSTUS models. Only scaffolds with a size greater than 2 kb were annotated.

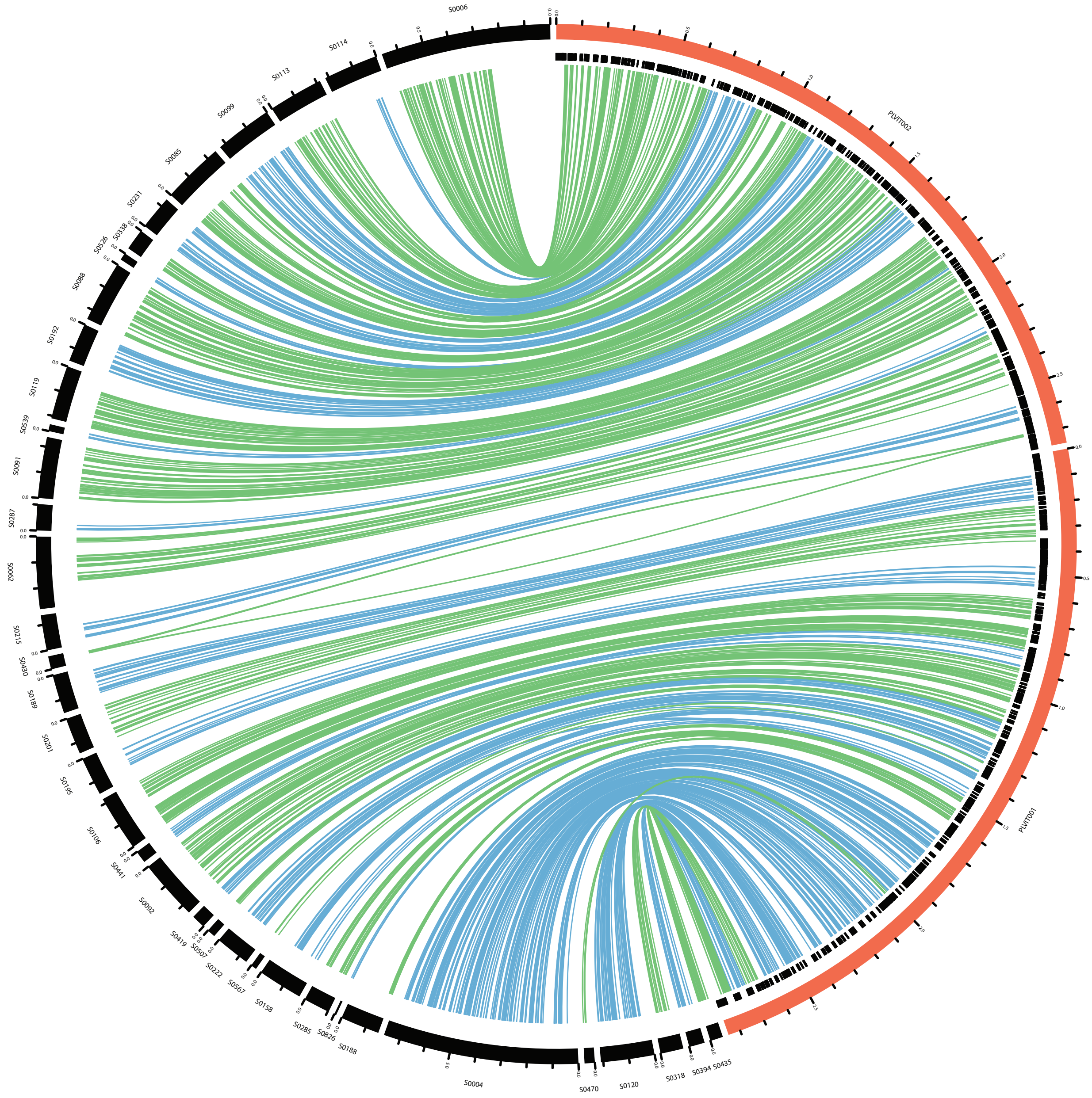

Supplementary figure 1 Comparison between the long-read and short-read assemblies of the *Pl. viticola* INRA-PV221 isolate. Only the results for the two biggest scaffolds of the long-read assembly are presented. Scaffolds of the long-read assembly are in red, those of the short-read assembly are in black, with tick marks each 100 kb. Repeat regions of the long-read assembly are represented as black boxes under the scaffolds. Links between scaffolds represents correspondance between genes. Links have different colors for alternating short-read scaffolds for clarity.

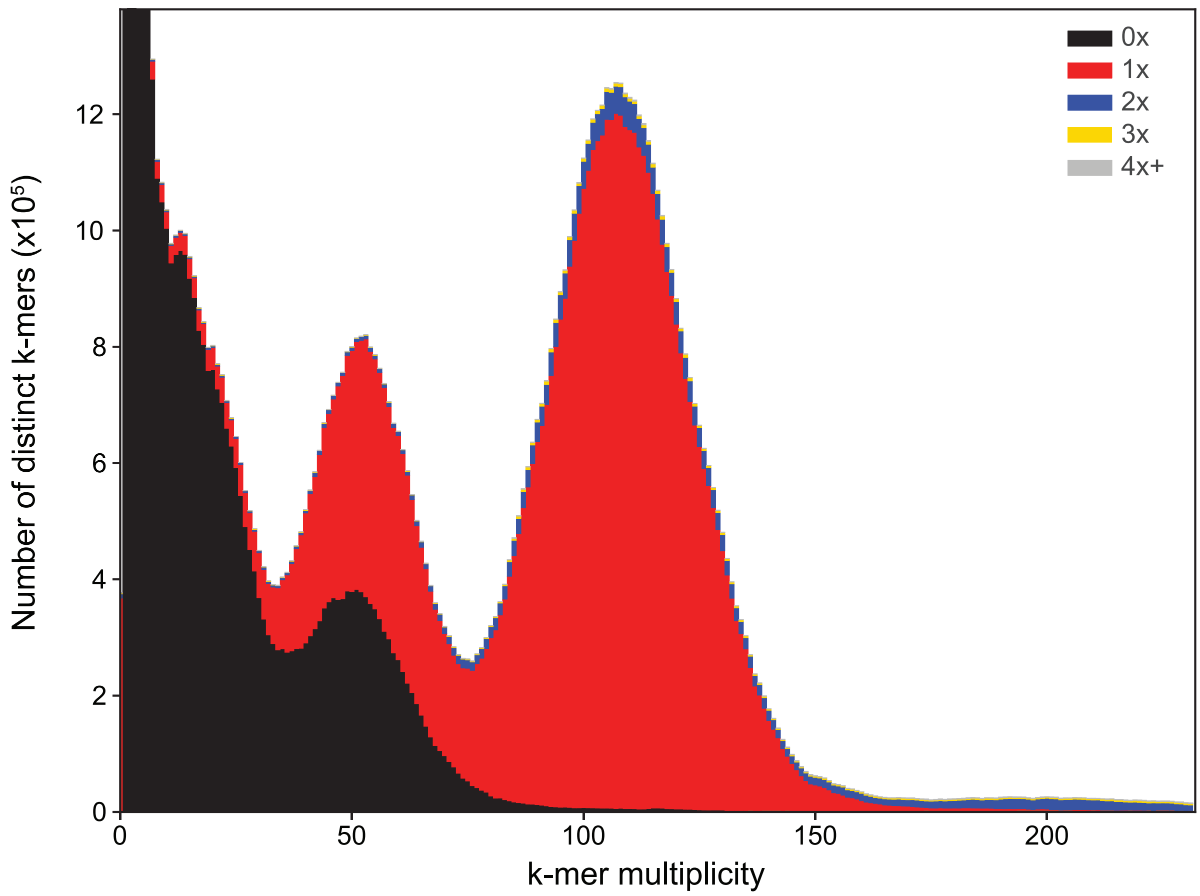

Supplementary figure 2 K-mer comparison between the corrected PacBio reads and the *Pl. viticola* genome assembly. The k-mer spectrum (k = 21) for the self-corrected PacBio reads is represented as a stacked histogram, with the differently colored areas corresponding to k-mers absent from the assembly (black), k-mers occuring only once in the assembly (red), k-mers occuring twice (blue), etc. Peaks around 55x and 110x respectively represent the heterozygous and homozygous content in the reads. K-mers from the homozygous content found twice in the assembly (in blue) could represent duplicated regions, i.e. heterozygous regions that have not been collapsed. K-mers from the heterozygous peak not found in the assembly (black area) represent the discarded heterozygous content, as expected for an haploid assembly.

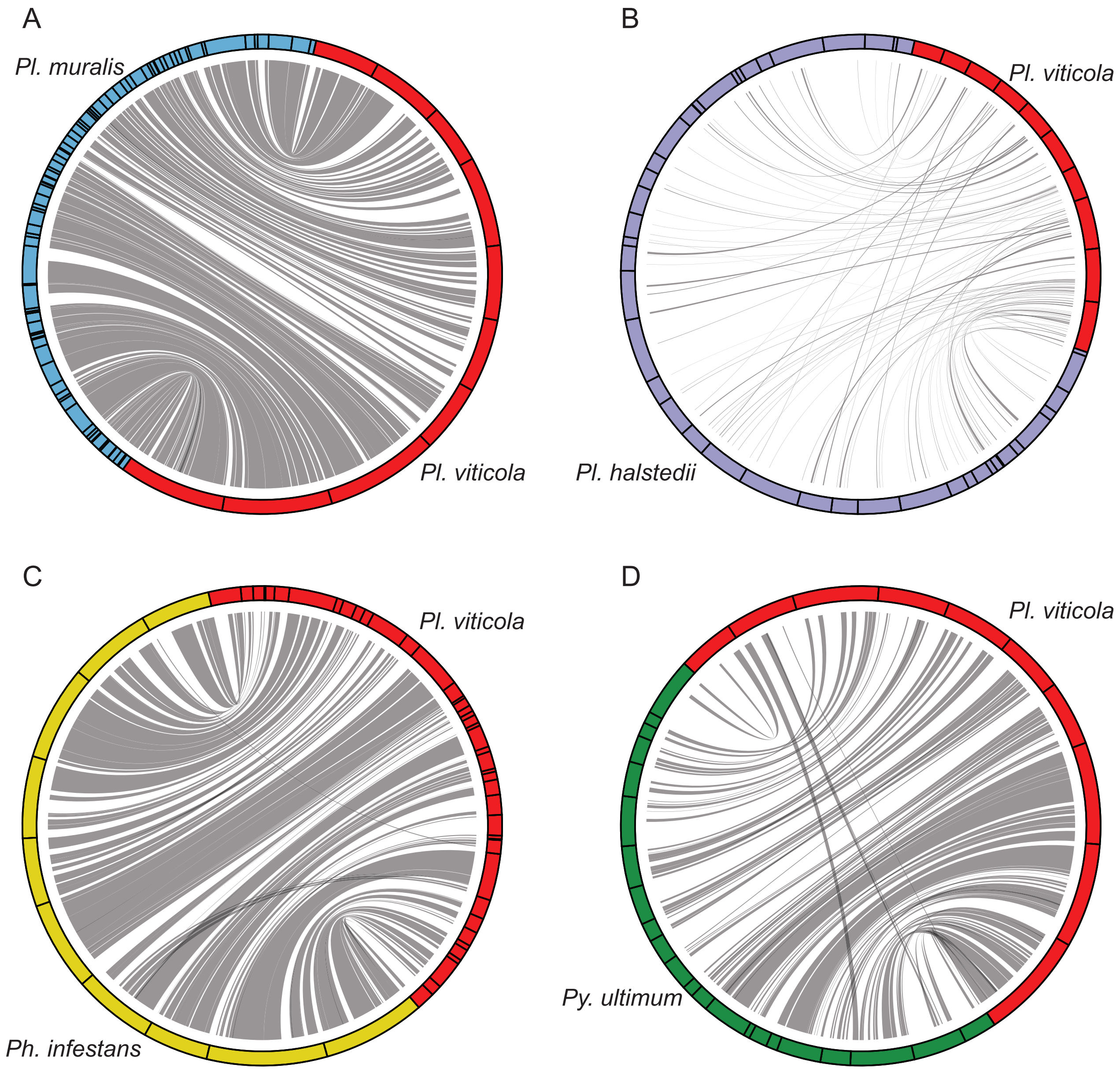

Supplementary figure 3 Synteny between *Pl. viticola* and (A) *Pl. muralis*, (B) *Pl. halstedii*, (C) *Ph. infestans* and (D) *Py. ultimum*. Syntenic regions are showed only for the ten largest *Pl. viticola* scaffolds, except for the comparison with *Ph. infestans*, where data is shown for the ten largest *Ph. infestans* scaffolds. Grey ribbons represent syntenic regions between genomes.

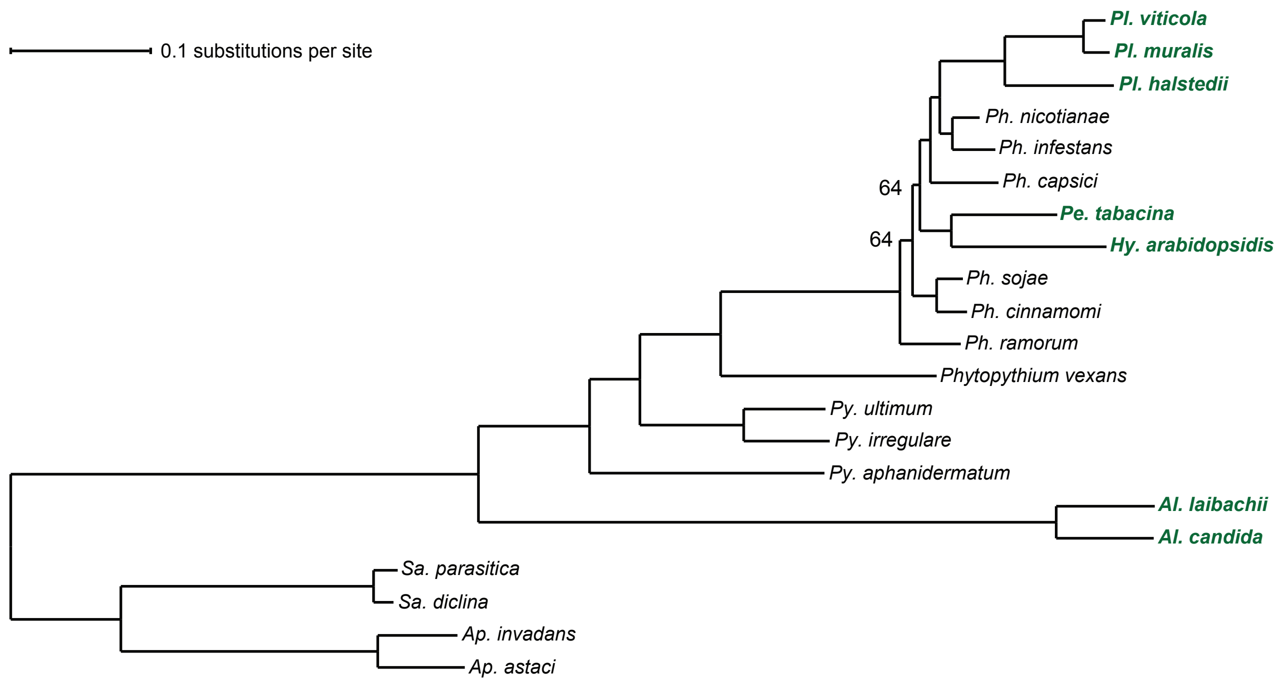

Supplementary figure 4 Phylogenetic relationships between Oomycete species. The maximum likelihood species tree was build using 144 single-copy orthologs. All nodes had a bootstrap support of 100% unless otherwise indicated. Biotrophic species names are shown in bold green typeface.

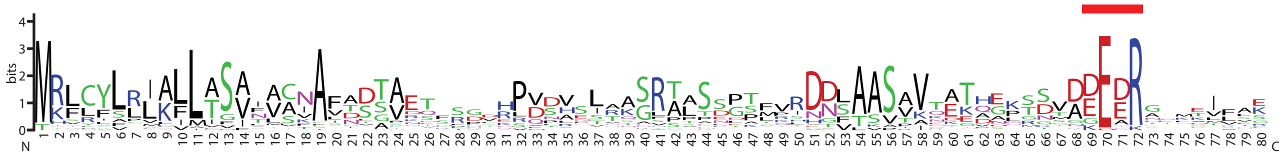

Supplementary figure 5 Sequence logo of *Pl. viticola* and *Pl. muralis* proteins with a WY motif but no RxLR signal. Proteins (38 sequences) were aligned with MAFFT and positions with more than 85% missing data (gaps) were discarded. The dEER motif common to all proteins is at positions 69-71 and is indicated by a red box.

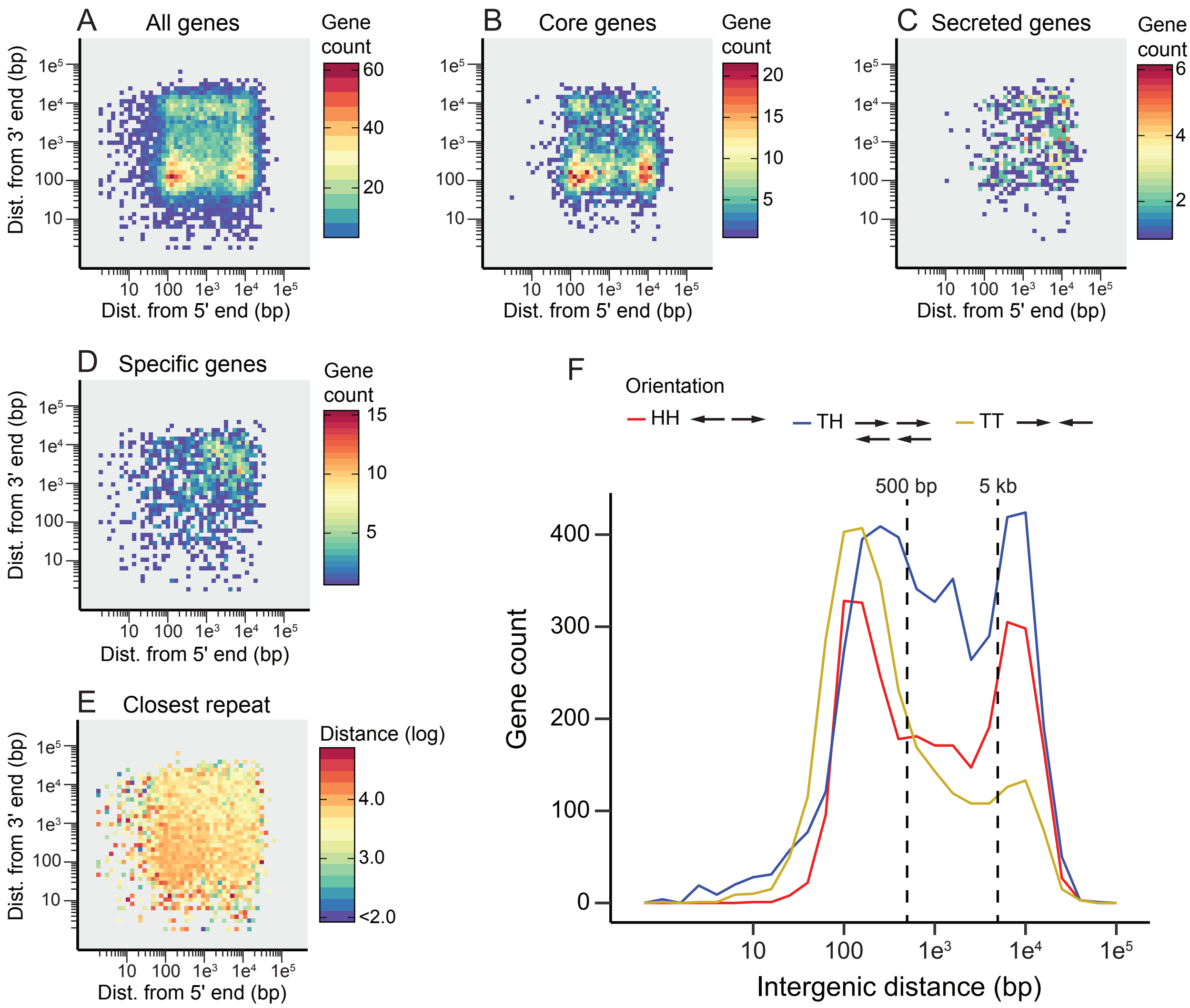

Supplementary figure 6 Local gene density and organization in *Pl. muralis*. Local gene density when considering (A) all genes, (B) core genes found in ortholog groups common to all Oomycetes, (C) genes encoding secreted proteins and (D) genes only found in *Pl. muralis* or in both *Pl. viticola* and *Pl. muralis*. The number of genes for two-dimensional (5' and 3') intergenic distance bins is represented as a heatmap. (E) Mean distance between genes and their closest repeat element, represented as a heatmap for two-dimensional intergenic distance bins. (F) Orientation and intergenic distance of adjacent gene pairs. The gene count for different intergenic distances has been plotted for adjacent gene pairs with a head-to-head (HH), tail-to-head (TH) and tail-to-tail (TT) orientation. Dashed vertical lines indicate the cut-offs used for the gene orientation analysis. Distances are on a log scale for all figures.

A

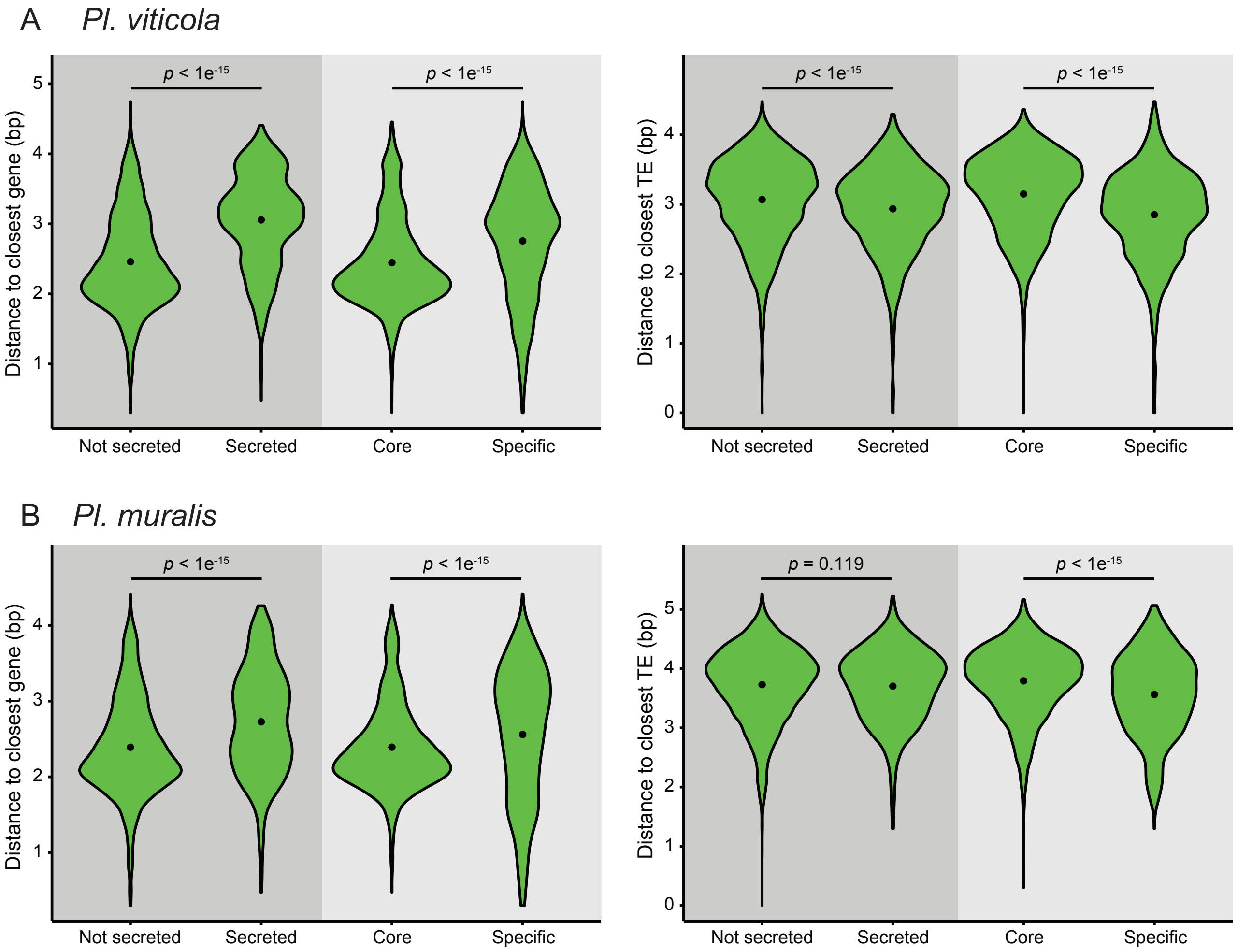

Supplementary figure 7 Distribution of distances to the closest gene and repeat sequence in (A) *Pl. viticola* and (B) *Pl. muralis* for different gene categories. Core genes were only found in orthogroups common to all oomycete species. Specific genes were only found in *Pl. viticola*/*Pl. muralis* or in both species. Average values are represented with a black dot. *P* values were computed using a Wilcoxon rank-sum test. Distances are on a log scale for all panels.

Supplementary table 1 Oomycete species and data origin

Species	Code	Source	Assembly version	Reference	Used for			
					Annotation	Orthology	GO enrichment for biotrophy	Branch-site tests
Albugo candida	ALCA	Ensembl	ASM107853v1	McMullan *et al.* 2015	X	X		
Albugo laibachii	ALLA	Ensembl	ENA1	Kemen *et al.* 2011	X	X		
Aphanomyces astaci	APAS	Ensembl	Apha_asta_APO3_V1	-		X	X	
Aphanomyces invadans	APIN	Ensembl	Apha_inva_NJM9701_V1	-		X	X	
Hyaloperonospora arabidopsidis	HYAR	Ensembl	HyaAraEmoy2_2.0	Baxter *et al.* 2010	X	X		
Peronospora tabacina	PETA	Corresponding author	968-J2_v1	Derevnina *et al.* 2015		X		
Phytophthora capsici	PHCA	JGI	LT1534 v11.0	Lamour *et al.* 2012	X	X		X
Phytophthora cinnamomi	PHCI	JGI	v1.0	-		X		
Phytophthora infestans	PHIN	Ensembl	ASM14294v1 (T30-4)	Haas *et al.* 2009	X	X	X	X
Phytophthora nicotianae	PHNI	Ensembl	ASM148298v1	Liu *et al.* 2016		X	X	X
Phytophthora ramorum	PHRA	JGI	v1.1	Tyler *et al.* 2006		X	X	
Phytophthora sojae	PHSO	Ensembl	P_sojae_V3_0	Tyler *et al.* 2006		X	X	
Plasmopara halstedii	PLHA	Senckenberg Data & Metadata Repository*	-	Sharma *et al.* 2015	X	X		X
Plasmopara muralis	PLMU			This study		X		X
Plasmopara viticola	PLVI			This study		X		X
Pythium aphanidermatum	PYAP	Ensembl	pag1_scaffolds_v1	Adhikari *et al.* 2013	X	X		
Pythium irregulare	PYIR	Ensembl	pir_scaffolds_v1	Adhikari *et al.* 2013		X		
Pythium ultimum	PYUL	Ensembl	pug	Lévesque *et al.* 2010	X	X	X	
Phytopythium vexans	PYVE	Ensembl	pve_scaffolds_v1	Adhikari *et al.* 2013		X		
Saprolegnia diclina	SADI	Ensembl	Sap_diclina_VS20_V1	-		X	X	
Saprolegnia parasitica	SAPA	Ensembl	ASM15154v2	Jiang *et al.* 2013	X	X	X	

Annotation: the proteomes of the species have been used as evidence for gene annotation. Orthology: proteomes used for the orthology analysis.
GO enrichment for biotrophy: the functional annotation of these species has been used for the GO enrichment analysis for ortholog groups absent in biotrophic species.
*http://dataportal-senckenberg.de/database/metacat/rsharma.26.4/bikf

Supplementary table 2 Assembly statistics for published *Pl. viticola* genome sequences

	JL-7-2 (Yin *et al.* 2017)	PvitFEM01 (Brilli *et al.* 2018)	INRA-PV221 (Dussert *et al.* 2016)	INRA-PV221 (this study)
Assembly size (Mb)	101.3	83.0	74.8	93.0
Total gap length	16.9 Mb	3.1 Mb	2.1 Mb	34.3 kb
Number of scaffolds (minimum size)	2,165 (500 pb)	57,336 (300 bp)	1,883 (1 kb)	358 (20 kb)
Max. scaffold size (kb)	806	132	763	2,850
N50 (size in kb / count)	172.3 / 172	4.1 / 3,733	180.6 / 130	706.5 / 38
% of repeat elements	25.60	NA	NA	37.34
BUSCO genome [S / D / F] (%)	66.6 [61.5 / 4.7 / 0.4]	61.1 [58.5 / 0.0 / 2.6]	92.8 [86.8 / 4.7 / 1.3]	95.7 [93.6 / 1.7 / 0.4]
Number of genes	17,014	38,298	NA	15,960
BUSCO proteome [S / D / F] (%)	65.8 [62.4 / 2.1 /1.3]	56.4 [53.0 / 0.4 / 3.0]	NA	97.4 [94.0 / 3.0 / 0.4]

NA: data not available

BUSCO analyses have been carried out with the Alveolata-Stramenopiles data set. S: single copy, D: duplicated, F: fragmented genes

Supplementary table 3 Type of repeat sequences in the *Pl. viticola* and *Pl. muralis* genomes

	Pl. viticola	*Pl. muralis*
Class I		
DIRS-like	1.69	4.36
LINES	6.62	10.17
LTR	42.93	61.61
LTR (non-autonomous derivatives)	35.53	0.77
Penelope-like	0.61	0.70
Others	0.00	4.90
Class II		
Crypton	0.00	4.36
Maverick	6.74	3.94
TIR	3.34	6.77
Others	0.99	0.82
No category	1.55	1.60

Repeat sequences are classified following Wicker *et al.* (2007), and numbers are percentages of repeat of a given type.

Supplementary table 4 Statistics of the synteny analysis between the genomes of *Pl. viticola* and four other oomycete species

	vs *Pl. muralis*	vs *Pl. halstedii*	vs *Ph. infestans*	vs *Py. ultimum*
Mean number of genes by syntenic block [range]	23 [5 – 130]	7 [5 – 21]	26 [5 – 242]	16 [5 – 141]
Mean syntenic block size in *Pl. viticola* [range] (kb)	132 [5 – 716]	38 [6 – 128]	172 [5 – 1,533]	130 [9 – 973]
Mean syntenic block size in compared species [range] (kb)	118 [5 – 686]	29 [6 – 153]	414 [5 – 2,007]	80 [3 – 618]
Total size of syntenic regions in *Pl. viticola* (Mb)	46.49	9.46	40.43	35.06
Total size of syntenic regions in compared species (Mb)	41.49	7.40	97.33	21.73
Total number of genes in syntenic blocks	7,900	1,184	6,136	4,379

Supplementary table 5 GO enrichment analysis for ortholog groups missing in oomycete biotrophic species

GO ID	GO term	Ontology	Annotated	Significant	Expected	*P* -values	Empirical *q* -values
GO:0006310	DNA recombination	BP	749	117	11.71	1.02E-81	0.00
GO:0015074	DNA integration	BP	778	118	12.16	6.26E-81	0.00
GO:0032217	riboflavin transporter activity	MF	28	28	0.39	7.56E-53	0.00
GO:0015171	amino acid transmembrane transporter activity	MF	403	54	5.63	7.53E-36	0.00
GO:0031419	cobalamin binding	MF	22	19	0.31	6.96E-33	0.00
GO:0030798	trans-aconitate 2-methyltransferase activity	MF	18	16	0.25	2.72E-28	0.00
GO:0005216	ion channel activity	MF	1057	63	14.76	9.95E-22	0.00
GO:0003884	D-amino-acid oxidase activity	MF	24	14	0.34	1.67E-20	0.00
GO:0046416	D-amino acid metabolic process	BP	24	14	0.38	7.45E-20	0.00
GO:0036361	racemase activity, acting on amino acids and derivatives	MF	17	12	0.24	2.96E-19	0.00
GO:0003677	DNA binding	MF	4254	135	59.38	4.71E-19	0.00
GO:0006508	proteolysis	BP	1381	70	21.58	4.95E-18	0.00
GO:0004367	glycerol-3-phosphate dehydrogenase [NAD+] activity	MF	27	13	0.38	1.18E-17	0.00
GO:0004494	methylmalonyl-CoA mutase activity	MF	9	9	0.13	1.94E-17	0.00
GO:0005089	Rho guanyl-nucleotide exchange factor activity	MF	28	13	0.39	2.17E-17	0.00
GO:0046168	glycerol-3-phosphate catabolic process	BP	26	13	0.41	2.48E-17	0.00
GO:0035023	regulation of Rho protein signal transduction	BP	28	13	0.44	8.68E-17	0.00
GO:0008705	methionine synthase activity	MF	11	9	0.15	1.04E-15	0.00
GO:0004114	3',5'-cyclic-nucleotide phosphodiesterase activity	MF	127	20	1.77	1.60E-15	0.00
GO:0003865	3-oxo-5-alpha-steroid 4-dehydrogenase activity	MF	9	8	0.13	1.24E-14	0.00
GO:0031151	histone methyltransferase activity (H3-K79 specific)	MF	130	19	1.81	3.33E-14	0.00
GO:0043531	ADP binding	MF	11	8	0.15	2.22E-13	0.00
GO:0000104	succinate dehydrogenase activity	MF	43	12	0.6	5.26E-13	0.00
GO:0019236	response to pheromone	BP	22	9	0.34	2.16E-11	0.00
GO:0005509	calcium ion binding	MF	2173	72	30.33	2.29E-11	0.00
GO:0009235	cobalamin metabolic process	BP	29	9	0.45	3.95E-10	0.00
GO:0016628	oxidoreductase activity, acting on the CH-CH group of donors, NAD or NADP as acceptor	MF	53	10	0.74	3.04E-09	0.00
GO:0016559	peroxisome fission	BP	40	9	0.63	9.25E-09	0.00
GO:0008202	steroid metabolic process	BP	58	10	0.91	2.13E-08	0.00
GO:0007154	cell communication	BP	2015	76	31.49	2.21E-08	0.00
GO:0019888	protein phosphatase regulator activity	MF	83	11	1.16	2.39E-08	0.00
GO:0071949	FAD binding	MF	154	14	2.15	4.20E-08	0.00
GO:0051920	peroxiredoxin activity	MF	63	9	0.88	2.34E-07	0.00
GO:0004842	ubiquitin-protein transferase activity	MF	794	29	11.08	3.70E-06	0.00
GO:0007186	G-protein coupled receptor signaling pathway	BP	93	9	1.45	1.56E-05	2.77E-04
GO:0005388	calcium-transporting ATPase activity	MF	82	8	1.14	2.01E-05	2.77E-04
GO:0042558	pteridine-containing compound metabolic process	BP	127	10	1.98	3.27E-05	6.74E-04
GO:0016849	phosphorus-oxygen lyase activity	MF	154	10	2.15	6.75E-05	1.71E-03
GO:0051726	regulation of cell cycle	BP	447	19	6.99	9.46E-05	2.05E-03
GO:0009190	cyclic nucleotide biosynthetic process	BP	155	10	2.42	1.74E-04	2.75E-03
GO:0009116	nucleoside metabolic process	BP	372	16	5.81	2.90E-04	4.63E-03
GO:0016042	lipid catabolic process	BP	245	12	3.83	5.24E-04	8.08E-03
GO:0015297	antiporter activity	MF	454	16	6.34	7.73E-04	1.10E-02
GO:0015103	inorganic anion transmembrane transporter activity	MF	337	13	4.7	1.04E-03	1.37E-02
GO:0007165	signal transduction	BP	1968	67	30.76	1.09E-03	1.41E-02
GO:0008138	protein tyrosine/serine/threonine phosphatase activity	MF	229	10	3.2	1.56E-03	1.82E-02
GO:0003777	microtubule motor activity	MF	926	25	12.93	1.58E-03	1.82E-02
GO:0004420	hydroxymethylglutaryl-CoA reductase (NADPH) activity	MF	17	3	0.24	1.59E-03	1.82E-02
GO:0010181	FMN binding	MF	363	13	5.07	2.02E-03	2.60E-02
GO:0016504	peptidase activator activity	MF	6	2	0.08	2.81E-03	3.54E-02
GO:0050982	detection of mechanical stimulus	BP	7	2	0.11	4.86E-03	6.64E-02
GO:0051287	NAD binding	MF	410	13	5.72	5.59E-03	8.00E-02

Ontology: BP, biological process; MF, molecular function. Annotated: total number of proteins mapped with the GO term. Significant: number of proteins absent from biotrophic species mapped with the GO term. Expected: expected number of proteins mapped with the GO term if randomly distributed. *P* -values: computed using the Fisher exact test. Empirical *q* -values: computed after 200 permutations.

Supplementary table 6 Number of flavoproteins in biotrophic and non-biotrophic oomycetes

Species	FAD binding	FMN binding	Total flavoproteins
Non-biotrophs			
Aphanomyces astaci	23	25	48
Aphanomyces invadans	17	25	42
Phytophthora infestans	23	43	66
Phytophthora nicotianae	21	44	65
Phytophthora ramorum	17	47	64
Phytophthora sojae	16	90	106
Pythium ultimum	12	46	58
Saprolegnia diclina	11	20	31
Saprolegnia parasitica	14	23	37
Mean	17.11	40.33	57.44
Biotrophs			
Albugo candida	3	8	11
Albugo laibachii	3	10	13
Hyaloperonospora arabidopsidis	6	15	21
Plasmopara halstedii	5	11	16
Plasmopara muralis	3	16	19
Plasmopara viticola	4	17	21
Mean	4.00	12.83	16.83

Supplementary table 7 KEGG annotation of *Ph. infestans* orthologs missing in biotrophic species

Gene	Definition	Pathways
PITG_00496	3',5'-cyclic-nucleotide phosphodiesterase	Purine metabolism
PITG_01804	5-methyltetrahydrofolate--homocysteine methyltransferase (methionine synthase)	Cysteine and methionine metabolism; Selenocompound metabolism; One carbon pool by folate; Metabolic pathways; Biosynthesis of secondary metabolites; Biosynthesis of amino acids
PITG_01808	methylmalonyl-CoA mutase	Valine, leucine and isoleucine degradation; Glyoxylate and dicarboxylate metabolism; Propanoate metabolism; Metabolic pathways; Carbon metabolism
PITG_02105	uracil phosphoribosyltransferase	Pyrimidine metabolism; Metabolic pathways
PITG_05696	glycerol-3-phosphate dehydrogenase (NAD+)	Glycerophospholipid metabolism; Biosynthesis of secondary metabolites
PITG_05701	glycerol-3-phosphate dehydrogenase (NAD+)	Glycerophospholipid metabolism; Biosynthesis of secondary metabolites
PITG_06147	nicotinamide mononucleotide adenylyltransferase	Nicotinate and nicotinamide metabolism; Metabolic pathways
PITG_08135	D-aspartate oxidase	Alanine, aspartate and glutamate metabolism; Peroxisome
PITG_10312	patatin-like phospholipase domain-containing protein 2	Glycerolipid metabolism; Metabolic pathways
PITG_13128	7-dehydrocholesterol reductase	Steroid biosynthesis; Metabolic pathways; Biosynthesis of secondary metabolites
PITG_18374	lysophosphatidylcholine acyltransferase / lyso-PAF acetyltransferase	Glycerophospholipid metabolism; Ether lipid metabolism; Metabolic pathways
PITG_18748	proteasome activator subunit 4	Proteasome
PITG_18972	sarcosine oxidase / L-pipecolate oxidase	Glycine, serine and threonine metabolism; Lysine degradation; Metabolic pathways; Peroxisome
PITG_21269	proteasome activator subunit 4	Proteasome
PITG_21426	Delta7-sterol 5-desaturase	Steroid biosynthesis; Metabolic pathways; Biosynthesis of secondary metabolites; Biosynthesis of antibiotics

Supplementary table 8 GO enrichment analysis for the secretome of *Pl. viticola*

GO ID	GO term	Ontology	Annotated	Significant	Expected	*P* -values	Empirical *q* -values
GO:0004252	serine-type endopeptidase activity	MF	130	70	7.48	6.10E-53	0.00
GO:0000272	polysaccharide catabolic process	BP	101	69	5.89	6.78E-33	0.00
GO:0004553	hydrolase activity, hydrolyzing O-glycosyl compounds	MF	176	99	10.13	1.19E-32	0.00
GO:0008810	cellulase activity	MF	41	30	2.36	5.17E-29	0.00
GO:0006508	proteolysis	BP	723	125	42.18	4.66E-27	0.00
GO:0030245	cellulose catabolic process	BP	28	23	1.63	1.80E-24	0.00
GO:0005975	carbohydrate metabolic process	BP	358	126	20.89	1.84E-20	0.00
GO:0030570	pectate lyase activity	MF	25	19	1.44	2.63E-19	0.00
GO:0030246	carbohydrate binding	MF	92	44	5.30	1.01E-18	0.00
GO:0030248	cellulose binding	MF	17	14	0.98	2.17E-15	0.00
GO:0030599	pectinesterase activity	MF	21	15	1.21	8.27E-15	0.00
GO:0042545	cell wall modification	BP	21	15	1.23	9.65E-15	0.00
GO:0004568	chitinase activity	MF	16	12	0.92	1.74E-12	0.00
GO:0006032	chitin catabolic process	BP	16	12	0.93	1.98E-12	0.00
GO:0009405	pathogenesis	BP	38	15	2.22	1.09E-09	0.00
GO:0016798	hydrolase activity, acting on glycosyl bonds	MF	199	110	11.46	2.20E-09	0.00
GO:0006952	defense response	BP	53	17	3.09	3.56E-09	0.00
GO:0016998	cell wall macromolecule catabolic process	BP	13	9	0.76	4.20E-09	0.00
GO:0009251	glucan catabolic process	BP	47	32	2.74	2.59E-07	0.00
GO:0004867	serine-type endopeptidase inhibitor activity	MF	8	6	0.46	8.99E-07	0.00
GO:1900004	negative regulation of serine-type endopeptidase activity	BP	8	6	0.47	9.66E-07	0.00
GO:0008422	beta-glucosidase activity	MF	23	9	1.32	2.57E-06	2.25E-04
GO:0003993	acid phosphatase activity	MF	25	9	1.44	5.79E-06	4.13E-04
GO:0008236	serine-type peptidase activity	MF	161	79	9.27	1.56E-05	4.13E-04
GO:0004650	polygalacturonase activity	MF	5	4	0.29	5.19E-05	5.94E-04
GO:0016755	transferase activity, transferring amino-acyl groups	MF	20	7	1.15	8.10E-05	7.62E-04
GO:0071555	cell wall organization	BP	27	19	1.58	1.38E-04	1.83E-03
GO:0008378	galactosyltransferase activity	MF	6	4	0.35	1.49E-04	1.95E-03
GO:0004869	cysteine-type endopeptidase inhibitor activity	MF	7	4	0.40	3.31E-04	3.87E-03
GO:0010951	negative regulation of endopeptidase activity	BP	15	10	0.88	3.31E-04	3.87E-03
GO:0016722	oxidoreductase activity, oxidizing metal ions	MF	12	5	0.69	3.51E-04	3.87E-03
GO:0003756	protein disulfide isomerase activity	MF	12	5	0.69	3.51E-04	3.87E-03
GO:0010383	cell wall polysaccharide metabolic process	BP	9	6	0.53	7.41E-04	6.75E-03
GO:0004601	peroxidase activity	MF	28	7	1.61	8.27E-04	7.43E-03
GO:0008233	peptidase activity	MF	551	119	31.72	9.01E-04	7.92E-03
GO:0045330	aspartyl esterase activity	MF	9	4	0.52	1.09E-03	1.00E-02
GO:0045490	pectin catabolic process	BP	9	4	0.53	1.14E-03	1.00E-02
GO:0034976	response to endoplasmic reticulum stress	BP	31	7	1.81	1.70E-03	2.02E-02
GO:0008061	chitin binding	MF	5	3	0.29	1.74E-03	2.02E-02
GO:0042124	1,3-beta-glucanosyltransferase activity	MF	5	3	0.29	1.74E-03	2.02E-02
GO:0034411	cell wall (1->3)-beta-D-glucan biosynthetic process	BP	5	3	0.29	1.81E-03	2.05E-02
GO:0004197	cysteine-type endopeptidase activity	MF	32	7	1.84	1.92E-03	2.10E-02
GO:0006493	protein O-linked glycosylation	BP	18	5	1.05	3.00E-03	3.16E-02
GO:0005507	copper ion binding	MF	20	5	1.15	4.68E-03	4.85E-02
GO:0072593	reactive oxygen species metabolic process	BP	13	4	0.76	5.36E-03	5.81E-02
GO:0098869	cellular oxidant detoxification	BP	38	7	2.22	5.72E-03	6.07E-02
GO:0044275	cellular carbohydrate catabolic process	BP	50	28	2.92	6.19E-03	6.29E-02
GO:0003824	catalytic activity	MF	5353	446	308.20	8.79E-03	9.54E-02
GO:0004181	metallocarboxypeptidase activity	MF	15	4	0.86	8.92E-03	9.54E-02

Ontology: BP, biological process; MF, molecular function. Annotated: total number of proteins mapped with the GO term. Significant: number of secreted proteins mapped with the GO term. Expected: expected number of proteins mapped with the GO term if randomly distributed. *P* -values: computed using the Fisher exact test. Empirical *q* -values: computed after 200 permutations.

Supplementary table 9 GO enrichment analysis for the secretome of *Pl. muralis*

GO ID	GO term	Ontology	Annotated	Significant	Expected	*P* -values	Empirical *q* -values
GO:0004553	hydrolase activity, hydrolyzing O-glycosyl compounds	MF	96	38	3.97	2.19E-22	0.00
GO:0005975	carbohydrate metabolic process	BP	223	62	10.78	7.09E-18	0.00
GO:0004252	serine-type endopeptidase activity	MF	74	24	3.06	5.75E-16	0.00
GO:0009405	pathogenesis	BP	21	14	1.01	2.23E-14	0.00
GO:0006952	defense response	BP	27	15	1.3	1.22E-13	0.00
GO:0006508	proteolysis	BP	437	60	21.12	1.74E-12	0.00
GO:0030245	cellulose catabolic process	BP	16	9	0.77	1.05E-08	0.00
GO:0030248	cellulose binding	MF	9	6	0.37	3.55E-07	0.00
GO:0004866	endopeptidase inhibitor activity	MF	7	5	0.29	2.27E-06	0.00
GO:0030246	carbohydrate binding	MF	42	15	1.74	4.07E-06	0.00
GO:0010951	negative regulation of endopeptidase activity	BP	7	5	0.34	4.90E-06	0.00
GO:0016722	oxidoreductase activity, oxidizing metal ions	MF	9	5	0.37	1.27E-05	0.00
GO:0004197	cysteine-type endopeptidase activity	MF	22	7	0.91	1.89E-05	0.00
GO:0016755	transferase activity, transferring amino-acyl groups	MF	22	7	0.91	1.89E-05	0.00
GO:0000272	polysaccharide catabolic process	BP	46	20	2.22	3.03E-05	0.00
GO:0008233	peptidase activity	MF	336	55	13.88	3.14E-05	0.00
GO:0008422	beta-glucosidase activity	MF	17	6	0.7	3.95E-05	2.90E-04
GO:0030599	pectinesterase activity	MF	14	5	0.58	1.70E-04	1.56E-03
GO:0042124	1,3-beta-glucanosyltransferase activity	MF	8	4	0.33	1.75E-04	1.56E-03
GO:0003993	acid phosphatase activity	MF	23	6	0.95	2.61E-04	3.00E-03
GO:0034411	cell wall (1->3)-beta-D-glucan biosynthetic process	BP	8	4	0.39	3.19E-04	3.00E-03
GO:0005507	copper ion binding	MF	16	5	0.66	3.47E-04	3.00E-03
GO:0042545	cell wall modification	BP	14	5	0.68	3.53E-04	3.00E-03
GO:0009251	glucan catabolic process	BP	31	14	1.5	4.31E-04	3.70E-03
GO:0044036	cell wall macromolecule metabolic process	BP	17	8	0.82	5.21E-04	4.73E-03
GO:0004601	peroxidase activity	MF	18	5	0.74	6.36E-04	5.99E-03
GO:0030570	pectate lyase activity	MF	5	3	0.21	6.55E-04	5.99E-03
GO:0016263	glycoprotein-N-acetylgalactosamine 3-beta-galactosyltransferase activity	MF	5	3	0.21	6.55E-04	5.99E-03
GO:0016267	O-glycan processing, core 1	BP	5	3	0.24	1.04E-03	1.04E-02
GO:0004568	chitinase activity	MF	6	3	0.25	1.27E-03	1.28E-02
GO:0006955	immune response	BP	6	3	0.29	2.00E-03	2.05E-02
GO:0006032	chitin catabolic process	BP	6	3	0.29	2.00E-03	2.05E-02
GO:0003756	protein disulfide isomerase activity	MF	15	4	0.62	2.71E-03	2.94E-02
GO:0010181	FMN binding	MF	16	4	0.66	3.49E-03	3.68E-02
GO:0034976	response to endoplasmic reticulum stress	BP	33	6	1.59	4.40E-03	4.54E-02
GO:0070011	peptidase activity, acting on L-amino acid peptides	MF	289	46	11.94	4.78E-03	4.96E-02
GO:0098869	cellular oxidant detoxification	BP	24	5	1.16	5.04E-03	5.08E-02
GO:0003824	catalytic activity	MF	3342	209	138.06	5.86E-03	5.68E-02
GO:0045330	aspartyl esterase activity	MF	10	3	0.41	6.74E-03	6.56E-02
GO:0072593	reactive oxygen species metabolic process	BP	9	3	0.43	7.53E-03	7.49E-02
GO:0016798	hydrolase activity, acting on glycosyl bonds	MF	108	41	4.46	7.74E-03	7.49E-02
GO:0010393	galacturonan metabolic process	BP	10	3	0.48	1.04E-02	9.40E-02
GO:0045490	pectin catabolic process	BP	10	3	0.48	1.04E-02	9.40E-02
GO:0045488	pectin metabolic process	BP	10	3	0.48	1.04E-02	9.40E-02

Ontology: BP, biological process; MF, molecular function. Annotated: total number of proteins mapped with the GO term. Significant: number of secreted proteins mapped with the GO term. Expected: expected number of proteins mapped with the GO term if randomly distributed. *P* -values: computed using the Fisher exact test. Empirical *q* -values: computed after 200 permutations.

Supplementary table 10 Results of the detection of candidate RxLR effector genes in *Pl. viticola* and *Pl. muralis*

	Pl. viticola	*Pl. muralis*
Regex/HMM only	58 (3)	21 (0)
BLASTP only	357 (312)	80 (62)
Regex/HMM + BLASTP	52 (25)	15 (5)
Iterative BLASTP	73 (1)	16 (0)
Total	540 (341)	132 (67)

Regex/HMM: search for regular expression using the Galaxy workflow of Cock *et al.* (2013).
BLASTP: BLASTP search at low stringency using an oomycete RxLR database.
Iterative BLASTP: BLASTP search at high stringency using the candidates detected by the Regex/HMM method as query.
Number of candidate effector genes showing structural homology to known RxLR effectors as detected by Phyre2 are shown in brackets.

Supplementary table 11 Orientation and intergenic distances for adjacent gene pairs in *Pl. viticola* and *Pl. muralis*

	Pl. viticola				*Pl. muralis*			
Intergenic distance	HH	TH	TT	*P*- value	HH	TH	TT	*P*- value
All	0.25	0.49	0.25	0.144	0.28	0.44	0.28	$< 1e^{-15}$
< 500 bp	0.22	0.44	0.34	$< 1e^{-15}$	0.24	0.37	0.38	$< 1e^{-15}$
> 5000 bp	0.32	0.51	0.18	$< 1e^{-15}$	0.36	0.49	0.16	$< 1e^{-15}$

HH: head-to-head orientation. TH: tail-to-head orientation. TT: tail-to-tail orientation. *P* -values were computed using a chi-square test comparing the observed proportions with the proportions expected by chance (0.25 for HH and TT, 0.50 for TH).

Supplementary table 12 *Pl. viticola* and *Pl. muralis* ortholog pairs with a dN/dS above 1

					Secreted		RxLR		GO terms		InterPro domains	
PVIT_gene	PMUR_gene	dN/dS	dN	dS	*Pl. viticola*	*Pl. muralis*	*Pl. viticola*	*Pl. muralis*	*Pl. viticola*	*Pl. muralis*	*Pl. viticola*	*Pl. muralis*
PVIT_0000327	PMUR_0010585	2.68	0.16	0.06	no	no	no	no				
**PVIT_0016683**	PMUR_0012182	2.27	0.08	0.04	no	no	no	no	protein kinase activity; ATP binding; protein phosphorylation	protein kinase activity; ATP binding; protein phosphorylation		
**PVIT_0003422**	PMUR_0003338	2.07	0.28	0.14	no	no	no	no				
**PVIT_0017530**	PMUR_0009546	2.04	0.37	0.18	yes	no	yes	no				
**PVIT_0012238**	PMUR_0003038	2.01	0.15	0.07	yes	no	yes	no				
PVIT_0017143	PMUR_0012479	2.00	0.17	0.08	no	no	no	no				
PVIT_0014100	PMUR_0004666	1.96	0.22	0.11	no	no	no	no				
**PVIT_0002472**	PMUR_0012195	1.92	0.18	0.09	yes	yes	no	no	carbohydrate binding		Jacalin-like lectin domain	Jacalin-like lectin domain
PVIT_0006091	PMUR_0001598	1.92	0.22	0.12	no	no	no	no	protein kinase activity; ATP binding; protein phosphorylation; kinase activity; phosphorylation			
**PVIT_0017049**	PMUR_0006507	1.91	0.14	0.07	no	no	no	no				
PVIT_0013491	PMUR_0010387	1.84	0.30	0.16	no	no	no	no				
PVIT_0005573	PMUR_0000810	1.76	0.21	0.12	no	no	no	no	calcium ion binding; protein binding		IQ motif, EF-hand binding site	
**PVIT_0010507**	PMUR_0008942	1.75	0.27	0.16	yes	yes	no	no				
**PVIT_0008293**	PMUR_0013073	1.73	0.21	0.12	yes	yes	no	no	serine-type endopeptidase activity; proteolysis; peptidase activity; membrane; integral component of membrane	serine-type endopeptidase activity; proteolysis	Serine proteases, trypsin domain; Peptidase S1A, chymotrypsin family; Peptidase S1, PA clan	Serine proteases, trypsin domain; Peptidase S1A, chymotrypsin family; Peptidase S1, PA clan
PVIT_0008070	PMUR_0008781	1.63	0.26	0.16	no	no	no	no				
**PVIT_0020870**	PMUR_0012887	1.55	0.17	0.11	no	no	no	no		nucleic acid binding		
PVIT_0010475	PMUR_0008920	1.51	0.18	0.12	no	no	no	no				
**PVIT_0006861**	PMUR_0002287	1.50	0.04	0.02	no	yes	no	no				
**PVIT_0017528**	PMUR_0013113	1.48	0.36	0.25	yes	yes	yes	yes				
**PVIT_0018321**	PMUR_0012509	1.46	0.16	0.11	no	no	no	no	nucleic acid binding; DNA binding	nucleic acid binding; DNA binding		
**PVIT_0012276**	PMUR_0003087	1.45	0.26	0.18	yes	yes	no	yes				
PVIT_0003436	PMUR_0012534	1.44	0.09	0.06	no	no	no	no	kinase activity; phosphorylation			
**PVIT_0013507**	PMUR_0010401	1.43	0.15	0.11	yes	yes	no	no				
**PVIT_0001143**	PMUR_0012041	1.42	0.19	0.13	no	no	no	no	nucleotide binding; nuclear exosome (RNase complex); nucleic acid binding; catalytic activity; exonuclease activity; intracellular; nucleobase-containing compound metabolic process; RNA processing; 3'-5' exonuclease activity; cellular metabolic process; nucleic acid phosphodiester bond hydrolysis	nucleotide binding; nuclear exosome (RNase complex); nucleic acid binding; catalytic activity; exonuclease activity; intracellular; nucleobase-containing compound metabolic process; RNA processing; 3'-5' exonuclease activity; cellular metabolic process; nucleic acid phosphodiester bond hydrolysis		
PVIT_0017527	PMUR_0012778	1.34	0.26	0.19	yes	yes	no	yes				
**PVIT_0003150**	PMUR_0004214	1.33	0.15	0.11	no	no	no	no	protein phosphorylation; motile cilium	protein phosphorylation		
**PVIT_0011823**	PMUR_0004836	1.32	0.16	0.12	no	yes	no	no	serine-type endopeptidase activity; proteolysis; peptidase activity	serine-type endopeptidase activity; proteolysis	Serine proteases, trypsin domain; Peptidase S1, PA clan	Serine proteases, trypsin domain; Peptidase S1, PA clan
**PVIT_0018318**	PMUR_0008586	1.31	0.04	0.03	yes	yes	yes	yes				
**PVIT_0013263**	PMUR_0012073	1.30	0.10	0.08	yes	no	yes	no				
**PVIT_0013728**	PMUR_0005019	1.29	0.04	0.03	no	no	no	no	membrane	membrane		
PVIT_0016143	PMUR_0008646	1.28	0.15	0.12	no	no	no	no				
PVIT_0006445	PMUR_0001691	1.26	0.44	0.35	no	no	no	no				
**PVIT_0006221**	PMUR_0002524	1.26	0.04	0.03	no	no	no	no	integral component of membrane	membrane		
PVIT_0012094	PMUR_0008697	1.24	0.52	0.42	no	no	no	no				
**PVIT_0003960**	PMUR_0000032	1.24	0.07	0.06	no	no	no	no				
**PVIT_0010797**	PMUR_0006150	1.23	0.16	0.13	no	yes	no	no				
**PVIT_0010792**	PMUR_0006148	1.22	0.19	0.16	yes	yes	no	no				
**PVIT_0001002**	PMUR_0011244	1.21	0.07	0.06	no	no	no	no	membrane; integral component of membrane			
**PVIT_0001929**	PMUR_0004106	1.20	0.13	0.11	yes	no	no	no				
**PVIT_0010451**	PMUR_0008115	1.20	0.30	0.25	no	no	no	no				
PVIT_0018773	PMUR_0008030	1.20	0.20	0.16	no	no	no	no				
PVIT_0027098	PMUR_0013178	1.20	0.02	0.02	no	no	no	no				
PVIT_0000387	PMUR_0005950	1.19	0.56	0.47	no	no	no	no	nucleic acid binding			
**PVIT_0021601**	PMUR_0012745	1.19	0.16	0.14	yes	yes	no	no				
**PVIT_0015335**	PMUR_0012557	1.18	0.31	0.26	yes	yes	yes	no				
PVIT_0013390	PMUR_0009670	1.18	0.24	0.20	no	no	no	no				
**PVIT_0016508**	PMUR_0006959	1.17	0.17	0.15	yes	yes	yes	yes				
**PVIT_0018732**	PMUR_0003335	1.17	0.21	0.18	no	no	no	no				

**PVIT_0001362**	PMUR_0003678	1.17	0.14	0.12	no	no	no	no				
PVIT_0003301	PMUR_0012893	1.16	0.30	0.26	no	no	no	no				
PVIT_0009061	PMUR_0010883	1.16	0.38	0.32	no	no	no	no	nucleic acid binding; nucleus; DNA integration	nucleus		Chromo/chromo shadow domain; Chromo domain; Chromo-like domain superfamily
**PVIT_0005694**	PMUR_0011772	1.16	0.23	0.20	no	no	no	no				
**PVIT_0019930**	PMUR_0012761	1.16	0.25	0.22	no	no	no	no	extracellular region; proteolysis; hydrolase activity			
**PVIT_0024609**	PMUR_0010001	1.15	0.27	0.24	no	no	no	no				
PVIT_0006028	PMUR_0011692	1.15	0.22	0.19	no	no	no	no				
PVIT_0003473	PMUR_0009677	1.14	0.21	0.19	no	no	no	no		nucleotide binding; ATP binding; hydrolase activity		P-loop containing nucleoside triphosphate hydrolase
**PVIT_0002228**	PMUR_0008345	1.13	0.06	0.05	no	no	no	no	tRNA modification; tRNA (guanine-N7-)-methyltransferase activity; RNA (guanine-N7)-methylation; tRNA methyltransferase complex	tRNA modification; tRNA (guanine-N7-)-methyltransferase activity; RNA (guanine-N7)-methylation; tRNA methyltransferase complex	tRNA (guanine-N-7) methyltransferase, Trmb type	tRNA (guanine-N-7) methyltransferase, Trmb type
**PVIT_0001383**	PMUR_0003688	1.13	0.14	0.13	yes	no	yes	no				
**PVIT_0010026**	PMUR_0012725	1.13	0.21	0.19	yes	yes	yes	yes				
**PVIT_0007026**	PMUR_0012532	1.13	0.08	0.07	yes	no	no	no	serine-type endopeptidase inhibitor activity; protein binding; proteolysis; peptidase activity; negative regulation of serine-type endopeptidase activity	protein binding; proteolysis; peptidase activity	Kazal domain	Kazal domain
**PVIT_0005735**	PMUR_0008108	1.13	0.17	0.15	yes	no	no	no				
**PVIT_0001577**	PMUR_0003648	1.12	0.25	0.22	no	no	no	no				
**PVIT_0001521**	PMUR_0003838	1.12	0.06	0.06	no	no	no	no				
PVIT_0020910	PMUR_0011234	1.10	0.17	0.16	no	no	no	no				
PVIT_0022528	PMUR_0007267	1.10	0.21	0.19	no	no	no	no				
**PVIT_0018709**	PMUR_0011974	1.09	0.19	0.18	yes	yes	yes	yes				
PVIT_0012353	PMUR_0004501	1.09	0.38	0.35	no	no	no	no				
**PVIT_0003385**	PMUR_0003939	1.08	0.14	0.13	no	yes	no	no	membrane; integral component of membrane	membrane; integral component of membrane		
**PVIT_0006586**	PMUR_0009062	1.08	0.34	0.32	yes	yes	no	no			CAP domain	CAP domain
PVIT_0016675	PMUR_0009646	1.08	0.11	0.10	no	no	no	no				
PVIT_0011285	PMUR_0012009	1.06	0.13	0.12	no	no	no	no				
**PVIT_0005562**	PMUR_0000823	1.06	0.17	0.16	no	no	no	no				
**PVIT_0018948**	PMUR_0011315	1.06	0.18	0.17	no	no	no	no	nucleic acid binding; DNA integration			
**PVIT_0017047**	PMUR_0009702	1.06	0.25	0.24	no	no	no	no				
**PVIT_0017162**	PMUR_0006259	1.05	0.06	0.06	no	no	no	no	protein phosphorylation	protein phosphorylation		
PVIT_0025603	PMUR_0012836	1.05	0.30	0.28	no	no	no	no				
**PVIT_0001524**	PMUR_0003841	1.05	0.08	0.07	no	no	no	no	membrane; integral component of membrane			
**PVIT_0022516**	PMUR_0011087	1.05	0.11	0.11	yes	yes	yes	yes				
**PVIT_0020918**	PMUR_0006134	1.05	0.12	0.11	yes	no	no	no				
**PVIT_0018905**	PMUR_0009659	1.04	0.14	0.13	yes	no	yes	no				
**PVIT_0005339**	PMUR_0001275	1.04	0.12	0.12	no	no	no	no	microtubule cytoskeleton organization; microtubule; microtubule binding			
**PVIT_0015820**	PMUR_0006188	1.04	0.14	0.14	no	yes	no	no				
**PVIT_0015154**	PMUR_0004184	1.03	0.24	0.23	yes	no	yes	no				
**PVIT_0001387**	PMUR_0003693	1.02	0.12	0.12	yes	yes	yes	yes				
**PVIT_0017599**	PMUR_0013161	1.02	0.10	0.10	yes	yes	no	no	polysaccharide catabolic process; hydrolase activity, hydrolyzing O-glycosyl compounds; extracellular region; carbohydrate metabolic process; metabolic process; cellulase activity; hydrolase activity; hydrolase activity, acting on glycosyl bonds; cellulose binding	polysaccharide catabolic process; hydrolase activity, hydrolyzing O-glycosyl compounds; carbohydrate metabolic process; metabolic process; cellulase activity; hydrolase activity; hydrolase activity, acting on glycosyl bonds	Glycoside hydrolase family 12; Glycoside hydrolase family 11/12; Concanavalin A-like lectin/glucanase domain superfamily	Phosphatidylinositol-specific phospholipase C, X domain; Glycoside hydrolase family 12; Glycoside hydrolase family 11/12; Concanavalin A-like lectin/glucanase domain superfamily
PVIT_0015339	PMUR_0008381	1.01	0.07	0.07	no	no	no	no				
**PVIT_0002656**	PMUR_0013156	1.01	0.13	0.13	no	no	no	no				
**PVIT_0011304**	PMUR_0007264	1.01	0.13	0.13	yes	no	yes	no				
**PVIT_0006307**	PMUR_0009093	1.01	0.11	0.11	yes	no	yes	no				
**PVIT_0003402**	PMUR_0003979	1.00	0.31	0.31	yes	yes	yes	yes				
**PVIT_0024814**	PMUR_0012891	1.00	0.19	0.19	yes	no	no	no	protein phosphorylation; membrane; integral component of membrane		HNH nuclease	
**PVIT_0012948**	PMUR_0001040	1.00	0.09	0.09	no	no	no	no				

Pl. viticola genes found expressed in the RNA-seq data are in bold.

Supplementary table 13 *Pl. viticola* genes with a significant signal in the branch-site test

Tested gene	Orthogroup	Align. size	Delta LRT	*P* -value	Q-value	Secreted	GO terms	InterPro domains
**PVIT_0006791**	Phyca11_562426, PITG_00022T0, KUF92812, Phal03539, PMUR_0004720, PVIT_0006791	969	149.74	1.98E-34	7.95E-31	no	drug transmembrane transport; drug transmembrane transporter activity; antiporter activity; membrane; transmembrane transporter activity; transmembrane transport	Multi antimicrobial extrusion protein
**PVIT_0007111**	Phyca11_7933, PITG_17333T0, KUF77828, Phal11456, PMUR_0011460, PVIT_0007111	1593	118.00	1.73E-27	3.48E-24	no	protein kinase activity; protein serine/threonine kinase activity; ATP binding; protein phosphorylation	Serine/threonine-protein kinase, active site; Protein kinase domain; Protein kinase-like domain superfamily
**PVIT_0005972**	Phyca11_123187, PITG_11886T0, KUF89757, Phal04063, PMUR_0009113, PVIT_0005972	969	94.54	2.40E-22	3.21E-19	no	protein binding; cell; glycerol ether metabolic process; protein disulfide oxidoreductase activity; cell redox homeostasis; oxidation-reduction process	Ubiquitin domain; PUB domain; Ubiquilin
**PVIT_0020511**	Phyca11_107403, PITG_10896T0, KUF79858, Phal09861, PMUR_0008422, PVIT_0020511	1446	90.60	1.76E-21	1.77E-18	no	kinase activity; phosphorylation	
**PVIT_0001212**	Phyca11_545532, PITG_02671T0, KUF87724, Phal06919, PMUR_0001253, PVIT_0001212	756	79.58	4.64E-19	3.72E-16	no	cAMP-dependent protein kinase complex; cAMP-dependent protein kinase regulator activity; cAMP binding; regulation of protein kinase activity	Cyclic nucleotide-binding, conserved site; Cyclic nucleotide-binding domain; RmlC-like jelly roll fold; Cyclic nucleotide-binding-like
**PVIT_0019913**	Phyca11_528570, PITG_22924T0, KUF86048, Phal07896, PMUR_0008817, PVIT_0019913	1074	62.00	3.44E-15	2.31E-12	no	membrane	
**PVIT_0003711**	Phyca11_505784, PITG_21524T0, KUF78485, Phal07365, PMUR_0000200, PVIT_0003711	1539	61.24	5.05E-15	2.90E-12	no	monooxygenase activity; iron ion binding; oxidoreductase activity, acting on paired donors, with incorporation or reduction of molecular oxygen; lyase activity; heme binding; oxidation-reduction process	Cytochrome P450, conserved site; Cytochrome P450; Cytochrome P450, E-class, group I
**PVIT_0001295**	Phyca11_115737, PITG_02802T0, KUF79445, Phal11391, PMUR_0000958, PVIT_0001295	885	60.73	6.54E-15	3.28E-12	no	membrane	Band 7 domain; Stomatin family
**PVIT_0013045**	Phyca11_7396, PITG_01416T0, KUF81499, Phal14135, PMUR_0001125, PVIT_0013045	1419	58.93	1.63E-14	7.28E-12	no	protein binding; cell; anatomical structure morphogenesis; tissue development; single-organism cellular process; animal organ development; establishment of localization in cell	Tetratricopeptide repeat-containing domain; Tetratricopeptide-like helical domain superfamily; Tetratricopeptide repeat
**PVIT_0009562**	Phyca11_9865, PITG_11573T0, KUF85728, Phal01751, PMUR_0002096, PVIT_0009562	1038	58.26	2.30E-14	9.22E-12	no	proteolysis; peptidase activity	Serine aminopeptidase, S33
**PVIT_0005945**	Phyca11_19592, PITG_11966T0, KUF85566, Phal08576, PMUR_0006626, PVIT_0005945	1683	55.19	1.09E-13	3.99E-11	no	ATP binding; cytoplasm; mitochondrion; protein refolding	Chaperonin Cpn60; Chaperonin Cpn60/TCP-1 family; GroEL-like apical domain superfamily; GroEL-like equatorial domain superfamily
**PVIT_0021795**	Phyca11_503134, PITG_19668T0, KUF79961, Phal09173, PMUR_0010414, PVIT_0021795	555	54.20	1.81E-13	6.07E-11	no	dihydrolipoyllysine-residue acetyltransferase activity; mitochondrial matrix; ribosome; pyruvate metabolic process; metabolic process; transferase activity; transferase activity, transferring acyl groups; pyruvate dehydrogenase complex	Biotin/lipoyl attachment; Single hybrid motif
**PVIT_0011454**	Phyca11_511108, PITG_04545T0, KUF79947, Phal08124, PMUR_0011394, PVIT_0011454	894	53.55	2.52E-13	7.79E-11	no	GTPase activity; GTP binding; ribosome biogenesis	GTP binding domain; GTP-binding protein, orthogonal bundle domain superfamily; P-loop containing nucleoside triphosphate hydrolase
**PVIT_0020495**	Phyca11_70774, PITG_19530T0, KUF82231, Phal13118, PMUR_0008410, PVIT_0020495	399	53.39	2.74E-13	7.86E-11	no	nucleus; protein heterodimerization activity	Transcription factor CBF/NF-Y/archaeal histone domain; Histone-fold

Tested gene	Orthogroup	Align. size	Delta LRT	*P* -value	Q-value	Secreted	GO terms	InterPro domains
**PVIT_0011740**	Phyca11_554424, PITG_21176T0, KUF78349, Phal04006, PMUR_0004934, PVIT_0011740	1770	44.88	2.10E-11	5.61E-09	no	protein import into nucleus, translocation; protein serine/threonine kinase activity; binding; cytoplasm; protein phosphorylation; NLS-bearing protein import into nucleus; ribosomal protein import into nucleus; intracellular protein transport; nuclear localization sequence binding; Ran GTPase binding; protein transporter activity; nuclear membrane; nuclear periphery	Importin-beta, N-terminal domain; Armadillo-like helical; Armadillo-type fold
**PVIT_0007405**	Phyca11_562095, PITG_02022T0, KUF94557, Phal00740, PMUR_0007974, PVIT_0007405	5097	44.13	3.07E-11	7.71E-09	no	structural molecule activity; binding; protein binding; intracellular protein transport; integral component of membrane; vesicle-mediated transport; clathrin coat of trans-Golgi network vesicle; clathrin coat of coated pit	Clathrin, heavy chain, linker, core motif; Clathrin, heavy chain; Tetratricopeptide-like helical domain superfamily; Armadillo-type fold; Clathrin heavy chain, N-terminal; Clathrin, heavy chain/VPS, 7-fold repeat; Clathrin, heavy chain, propeller repeat
**PVIT_0006426**	Phyca11_506258, PITG_12916T0, KUF84984, Phal06271, PMUR_0011524, PVIT_0006426	1053	43.22	4.89E-11	1.16E-08	yes	cysteine-type endopeptidase activity; extracellular space; lysosome; proteolysis; cysteine-type peptidase activity; proteolysis involved in cellular protein catabolic process	Cysteine peptidase, cysteine active site; Cysteine peptidase, histidine active site; Cysteine peptidase, asparagine active site; Peptidase C1A, papain C-terminal; Peptidase C1A
**PVIT_0011623**	Phyca11_508456, PITG_13718T0, KUF90555, Phal11080, PMUR_0011240, PVIT_0011623	1239	39.55	3.19E-10	7.13E-08	no	copper ion transmembrane transporter activity; integral component of membrane; metal ion transport; copper ion transmembrane transport; metal ion binding	Heavy metal-associated domain, HMA; Ctr copper transporter
**PVIT_0011236**	Phyca11_511517, PITG_16916T0, KUF84073, Phal10179, PMUR_0012427, PVIT_0011236	1008	38.11	6.69E-10	1.41E-07	no	protein kinase activity; protein serine/threonine kinase activity; ATP binding; protein phosphorylation	Protein kinase, ATP binding site; Protein kinase domain; Serine-threonine/tyrosine-protein kinase, catalytic domain; Protein kinase-like domain superfamily
**PVIT_0017234**	Phyca11_9084, PITG_04000T0, KUF77695, Phal06372, PMUR_0001948, PVIT_0017234	990	35.35	2.76E-09	5.55E-07	no	protein binding	WD40 repeat, conserved site; WD40-repeat-containing domain; WD40/YVTN repeat-like-containing domain superfamily; WD40 repeat; G-protein beta WD-40 repeat
**PVIT_0017715**	Phyca11_550917, PITG_15026T0, KUF84755, Phal02249, PMUR_0012296, PVIT_0017715	2442	34.50	4.27E-09	8.17E-07	no	protein import into nucleus, translocation; binding; cytoplasm; NLS-bearing protein import into nucleus; ribosomal protein import into nucleus; nuclear localization sequence binding; protein transporter activity; nuclear membrane; nuclear periphery	Armadillo-like helical; Armadillo-type fold
**PVIT_0011847**	Phyca11_38642, PITG_13615T0, KUF82271, Phal04750, PMUR_0004845, PVIT_0011847	726	31.62	1.87E-08	3.42E-06	no	rRNA modification; rRNA (adenine-N6,N6-)-dimethyltransferase activity; cytoplasm; rRNA methyltransferase activity; rRNA methylation	Ribosomal RNA adenine methylase transferase, conserved site; Ribosomal RNA adenine methylase transferase, N-terminal; Ribosomal RNA adenine methyltransferase KsgA/Erm
**PVIT_0013056**	Phyca11_70439, PITG_01448T0, KUF81503, Phal06276, PMUR_0006342, PVIT_0013056	384	29.23	6.43E-08	1.12E-05	no	calcium ion binding	EF-Hand 1, calcium-binding site; EF-hand domain; EF-hand domain pair
**PVIT_0017784**	Phyca11_7234, PITG_17975T0, KUF79813, Phal00359, PMUR_0007002, PVIT_0017784	1161	28.72	8.37E-08	1.40E-05	no	protein binding; transferase activity; transferase activity, transferring glycosyl groups	GAF domain
**PVIT_0001644**	Phyca11_116367, PITG_00397T0, KUF78113, Phal01183, PMUR_0000545, PVIT_0001644	2541	26.73	2.34E-07	3.76E-05	no	formation of translation preinitiation complex; RNA binding; translation initiation factor activity; protein binding; eukaryotic translation initiation factor 3 complex; translational initiation; regulation of translational initiation; eukaryotic 43S preinitiation complex; translation initiation factor binding; eukaryotic 48S preinitiation complex	Proteasome component (PCI) domain; Eukaryotic translation initiation factor 3 subunit C, N-terminal domain; ArsR-like helix-turn-helix domain; Eukaryotic translation initiation factor 3 subunit C
**PVIT_0015346**	Phyca11_21290, PITG_07455T0, KUF88067, Phal01518, PMUR_0008383, PVIT_0015346	3525	26.64	2.45E-07	3.79E-05	no	nucleotide binding; magnesium ion binding; phospholipid-translocating ATPase activity; ATP binding; plasma membrane; phospholipid transport; integral component of membrane; phospholipid translocation; metal ion binding	P-type ATPase; P-type ATPase, subfamily IV; P-type ATPase, A domain superfamily; HAD superfamily; P-type ATPase, cytoplasmic domain N; P-type ATPase, phosphorylation site
**PVIT_0018929**	Phyca11_537183, PITG_11789T0, KUF82256, Phal01544, PMUR_0004550, PVIT_0018929	693	26.34	2.86E-07	4.10E-05	no	proton transport; vacuolar proton-transporting V-type ATPase complex; hydrolase activity, acting on acid anhydrides, catalyzing transmembrane movement of substances; transmembrane transport	Coiled-coil domain containing protein 109, C-terminal

Tested gene	Orthogroup	Align. size	Delta LRT	*P*-value	Q-value	Secreted	GO terms	InterPro domains
**PVIT_0019167**	Phyca11_547133, PITG_08661T0, KUG02176, Phal08958, PMUR_0011983, PVIT_0019167	1476	26.38	2.81E-07	4.10E-05	no	catalytic activity; RNA modification; transferase activity; protein kinase regulator activity; methylthiotransferase activity; tRNA methylthiolation; macromolecule modification; regulation of protein kinase activity; iron-sulfur cluster binding; 4 iron, 4 sulfur cluster binding	Methylthiotransferase, conserved site; Elp3/MiaB/NifB; Radical SAM; Methylthiotransferase, N-terminal; Methylthiotransferase; Radical SAM, alpha/beta horseshoe
**PVIT_0018786**	Phyca11_504262, PITG_19871T0, KUF80558, Phal01578, PMUR_0006571, PVIT_0018786	2388	26.05	3.33E-07	4.62E-05	no	ATP binding; hydrolase activity; cell division	ATPase, AAA-type, conserved site; CDC48, N-terminal subdomain; AAA+ ATPase domain; ATPase, AAA-type, core; CDC48, domain 2; Vps4 oligomerisation, C-terminal; AAA ATPase, CDC48 family; Aspartate decarboxylase-like domain superfamily; P-loop containing nucleoside triphosphate hydrolase
**PVIT_0001977**	Phyca11_509876, PITG_05224T0, KUG01522, Phal04316, PMUR_0007898, PVIT_0001977	423	25.01	5.71E-07	7.64E-05	no	microtubule motor activity; protein binding; ATP binding; kinesin complex; microtubule-based movement; microtubule binding; ATPase activity	Kinesin motor domain; SMAD/FHA domain superfamily; P-loop containing nucleoside triphosphate hydrolase
**PVIT_0012980**	Phyca11_534012, PITG_01330T0, KUF81212, Phal11222, PMUR_0001069, PVIT_0012980	1365	24.35	8.05E-07	1.04E-04	no	integral component of membrane; transferase activity, transferring acyl groups	Membrane bound O-acyl transferase, MBOAT
**PVIT_0003670**	Phyca11_564754, PITG_15837T0, KUF80592, Phal08051, PMUR_0004283, PVIT_0003670	1401	23.47	1.27E-06	1.59E-04	no	ATP binding; nucleus	SNF2-related, N-terminal domain; Chromo/chromo shadow domain; Helicase, C-terminal; Helicase superfamily 1/2, ATP-binding domain; Chromo domain; Chromo-like domain superfamily; P-loop containing nucleoside triphosphate hydrolase
**PVIT_0010101**	Phyca11_542607, PITG_02114T0, KUF76904, Phal04605, PMUR_0001777, PVIT_0010101	1383	23.14	1.51E-06	1.83E-04	no	aminopeptidase activity; proteolysis; metallopeptidase activity; zinc ion binding	Peptidase M18; Peptidase M18, domain 2
**PVIT_0018284**	Phyca11_34281, PITG_11759T0, KUF76521, Phal08738, PMUR_0009478, PVIT_0018284	1677	22.68	1.92E-06	2.27E-04	no	protein binding	Ankyrin repeat-containing domain; Ankyrin repeat
**PVIT_0000878**	Phyca11_525004, PITG_02268T0, KUF88600, Phal00471, PMUR_0004458, PVIT_0000878	825	22.34	2.28E-06	2.62E-04	no	integral component of membrane	
**PVIT_0016179**	Phyca11_561150, PITG_15735T0, KUF73665, Phal06406, PMUR_0002604, PVIT_0016179	1698	21.45	3.63E-06	3.94E-04	no	protein kinase activity; protein serine/threonine kinase activity; protein binding; ATP binding; nucleus; cytoplasm; protein phosphorylation; intracellular signal transduction	Protein kinase, ATP binding site; Protein kinase domain; AGC-kinase, C-terminal; Pleckstrin homology domain; Ankyrin repeat-containing domain; Protein kinase-like domain superfamily; PH-like domain superfamily; Ankyrin repeat
**PVIT_0019500**	Phyca11_533155, PITG_08734T0, KUF75663, Phal04137, PMUR_0002790, PVIT_0019500	2757	21.46	3.61E-06	3.94E-04	no	ion channel activity; voltage-gated chloride channel activity; chloride transport; membrane; integral component of membrane; ion transmembrane transport; transmembrane transport; regulation of anion transmembrane transport	CBS domain; Chloride channel, voltage gated; Chloride channel, core
**PVIT_0019185**	Phyca11_113258, PITG_08676T0, KUG02150, Phal11229, PMUR_0010308, PVIT_0019185	933	20.80	5.11E-06	5.40E-04	no	proteasome complex; protein binding; nucleus; cytosol; glycolytic process; N-terminal protein myristoylation; protein targeting to vacuole; fatty acid beta-oxidation; water transport; hyperosmotic response; Golgi organization; response to temperature stimulus; photomorphogenesis; photorespiration; kinase activity; proteasome-mediated ubiquitin-dependent protein catabolic process; proteasome assembly; response to cadmium ion; response to misfolded protein	JAB1/MPN/MOV34 metalloenzyme domain; Rpn11/EIF3F, C-terminal
**PVIT_0006624**	Phyca11_571282, PITG_11102T0, KUF76508, Phal02941, PMUR_0003642, PVIT_0006624	1812	20.68	5.43E-06	5.59E-04	no	protein kinase activity; protein serine/threonine kinase activity; ATP binding; protein phosphorylation	Serine/threonine-protein kinase, active site; Protein kinase, ATP binding site; Cyclic nucleotide-binding, conserved site; Cyclic nucleotide-binding domain; Protein kinase domain; AGC-kinase, C-terminal; Protein kinase-like domain superfamily; RmlC-like jelly roll fold; Cyclic nucleotide-binding-like

Tested gene	Orthogroup	Align. size	Delta LRT	*P* -value	Q-value	Secreted	GO terms	InterPro domains
**PVIT_0009795**	Phyca11_534373, PITG_07252T0, KUF76130, Phal00703, PMUR_0009200, PVIT_0009795	327	20.63	5.57E-06	5.59E-04	no	synaptonemal complex; single-stranded DNA binding; ATP binding; chiasma; mismatch repair; ATPase activity; MutLalpha complex	Histidine kinase/HSP90-like ATPase; MutL, C-terminal, dimerisation
**PVIT_0010806**	Phyca11_19812, PITG_07902T0, KUF78648, Phal01953, PMUR_0006158, PVIT_0010806	1365	20.42	6.22E-06	6.10E-04	no	nucleus	Chromo/chromo shadow domain; Chromo domain; Chromo-like domain superfamily
**PVIT_0001083**	Phyca11_569294, PITG_02550T0, KUF79783, Phal02833, PMUR_0005250, PVIT_0001083	4470	20.14	7.19E-06	6.72E-04	no	transcription factor activity, sequence-specific DNA binding; regulation of transcription, DNA-templated	
**PVIT_0006127**	Phyca11_527201, PITG_05560T0, KUF76730, Phal07792, PMUR_0001567, PVIT_0006127	1773	20.16	7.11E-06	6.72E-04	no	integral component of membrane	Transmembrane protein TqsA-like
**PVIT_0011106**	Phyca11_552564, PITG_16386T0, KUF83907, Phal00431, PMUR_0002397, PVIT_0011106	297	19.63	9.41E-06	8.59E-04	no	isomerase activity	BolA protein
**PVIT_0022488**	Phyca11_510798, PITG_08199T0, KUG01097, Phal02854, PMUR_0006243, PVIT_0022488	771	19.37	1.08E-05	9.60E-04	no	transcription factor activity, sequence-specific DNA binding; nucleus; regulation of transcription, DNA-templated; sequence-specific DNA binding	Heat shock factor (HSF)-type, DNA-binding; ArsR-like helix-turn-helix domain; Heat shock transcription factor family
**PVIT_0019045**	Phyca11_48915, PITG_23178T0, KUF89226, Phal07228, PMUR_0007287, PVIT_0019045	432	18.98	1.32E-05	1.16E-03	no	protein binding; proteolysis; peptidase activity; membrane; integral component of membrane	Kazal domain
**PVIT_0010430**	Phyca11_571966, PITG_09965T0, KUF94296, Phal07840, PMUR_0008135, PVIT_0010430	2079	18.83	1.43E-05	1.22E-03	no	intracellular protein transport; ER to Golgi vesicle-mediated transport; zinc ion binding; COPII vesicle coat	von Willebrand factor, type A; Zinc finger, Sec23/Sec24-type; Sec23/Sec24, trunk domain; Sec23/Sec24, helical domain; Gelsolin-like domain; Sec23/Sec24 beta-sandwich
**PVIT_0019353**	Phyca11_551426, PITG_19608T0, KUF88578, Phal00838, PMUR_0005105, PVIT_0019353	432	18.79	1.46E-05	1.22E-03	no	cleavage involved in rRNA processing; maturation of LSU-rRNA; RNA binding; nucleolus; translation; viral nucleocapsid; cytosolic large ribosomal subunit; rRNA pseudouridine synthesis; snRNA pseudouridine synthesis; box H/ACA snoRNP complex; box H/ACA snoRNA binding	Ribosomal protein L7Ae/L30e/S12e/Gadd45; H/ACA ribonucleoprotein complex, subunit Nhp2, eukaryote; Ribosomal protein L7Ae/L8/Nhp2 family
**PVIT_0021040**	Phyca11_506714, PITG_13041T0, KUF76086, Phal06340, PMUR_0006775, PVIT_0021040	2190	18.45	1.74E-05	1.43E-03	no	DNA binding; transcription elongation from RNA polymerase II promoter; histone modification; Cdc73/Paf1 complex; ligase activity	Plus-3 domain
**PVIT_0011277**	Phyca11_130345, PITG_19647T0, KUF84328, Phal07232, PMUR_0011200, PVIT_0011277	1359	17.71	2.58E-05	2.07E-03	no	nucleotide binding; protein kinase activity; ATP binding; protein phosphorylation; membrane; integral component of membrane; kinase activity; phosphorylation	Meiosis-specific nuclear structural protein 1
**PVIT_0000670**	Phyca11_8263, PITG_03474T0, KUG02199, Phal03009, PMUR_0000248, PVIT_0000670	972	17.58	2.75E-05	2.13E-03	no	integral component of membrane	Rab-GTPase-TBC domain
**PVIT_0018286**	Phyca11_503414, PITG_11755T0, KUF94440, Phal11255, PMUR_0010817, PVIT_0018286	1257	17.59	2.74E-05	2.13E-03	yes		
**PVIT_0009363**	Phyca11_503496, PITG_03788T0, KUF85578, Phal01185, PMUR_0007546, PVIT_0009363	738	17.30	3.19E-05	2.42E-03	no		
**PVIT_0001841**	Phyca11_570517, PITG_00634T0, KUF76976, Phal04188, PMUR_0003890, PVIT_0001841	2142	16.68	4.43E-05	3.30E-03	no	microtubule cytoskeleton organization; spindle pole; equatorial microtubule organizing center; gamma-tubulin complex; structural constituent of cytoskeleton; centrosome; microtubule organizing center; spindle pole body; microtubule nucleation; meiotic nuclear division; cytoplasmic microtubule organization; gamma-tubulin binding; microtubule minus-end binding; centrosome duplication; interphase microtubule nucleation by interphase microtubule organizing center; mitotic spindle assembly	Gamma-tubulin complex component protein
**PVIT_0004681**	Phyca11_5374, PITG_14971T0, KUF76285, Phal04784, PMUR_0011370, PVIT_0004681	2307	16.63	4.54E-05	3.32E-03	no	protein kinase activity; ATP binding; protein phosphorylation; membrane; kinase activity; phosphorylation	Protein kinase domain; Protein kinase-like domain superfamily

Tested gene	Orthogroup	Align. size	Delta LRT	*P* -value	Q-value	Secreted	GO terms	InterPro domains
**PVIT_0001940**	Phyca11_118922, PITG_00748T0, KUF96721, Phal12777, PMUR_0004112, PVIT_0001940	747	14.95	1.10E-04	7.92E-03	no	maturation of SSU-rRNA from tricistronic rRNA transcript (SSU-rRNA, 5.8S rRNA, LSU-rRNA); binding; snoRNA binding; 90S preribosome; small-subunit processome; t-UTP complex; positive regulation of transcription from RNA polymerase I promoter	BP28, C-terminal domain; U3 small nucleolar RNA-associated protein 10; Armadillo-like helical; Armadillo-type fold
**PVIT_0012672**	Phyca11_546135, PITG_13257T0, KUF80953, Phal03465, PMUR_0010352, PVIT_0012672	879	14.81	1.19E-04	8.37E-03	no	translation initiation factor activity; translational initiation	MaoC-like domain
**PVIT_0016699**	Phyca11_553199, PITG_21150T0, KUF78371, Phal08237, PMUR_0010053, PVIT_0016699	1422	14.45	1.44E-04	9.98E-03	no	transport; integral component of membrane	Major facilitator superfamily domain; Folate-biopterin transporter
**PVIT_0018593**	Phyca11_511666, PITG_06131T0, KUF87355, Phal04830, PMUR_0004484, PVIT_0018593	639	14.23	1.62E-04	1.10E-02	no	integral component of membrane; posttranslational protein targeting to membrane, translocation; Sec62/Sec63 complex	Translocation protein Sec66
**PVIT_0003408**	Phyca11_13754, PITG_05778T0, KUF93709, Phal00231, PMUR_0010404, PVIT_0003408	1722	13.95	1.87E-04	1.25E-02	no	protein binding	WD40 repeat, conserved site; WD40-repeat-containing domain; WD40/YVTN repeat-like-containing domain superfamily; WD40 repeat; G-protein beta WD-40 repeat
**PVIT_0009348**	Phyca11_539800, PITG_03792T0, KUF85576, Phal14413, PMUR_0007554, PVIT_0009348	1302	13.69	2.16E-04	1.42E-02	no	RNA binding; translation initiation factor activity; catalytic activity; cytoplasm; translational initiation; metabolic process; 2,3-bisphosphoglycerate-independent phosphoglycerate mutase activity; metal ion binding	Eukaryotic translation initiation factor 4E (eIF-4E), conserved site; Metalloenzyme; Translation Initiation factor eIF- 4e; 2,3-bisphosphoglycerate-independent phosphoglycerate mutase; Alkaline phosphatase-like, alpha/beta/alpha; Alkaline-phosphatase-like, core domain superfamily; Translation Initiation factor eIF- 4e-like
**PVIT_0007471**	Phyca11_532861, PITG_10152T0, KUF83836, Phal14326, PMUR_0001376, PVIT_0007471	612	13.48	2.41E-04	1.56E-02	no	calcium ion binding; integral component of membrane	EF-Hand 1, calcium-binding site; EF-hand domain; EF-hand domain pair
**PVIT_0002178**	Phyca11_527718, PITG_05274T0, KUF97665, Phal03178, PMUR_0008321, PVIT_0002178	2754	13.41	2.50E-04	1.59E-02	no	binding; cytoplasm; protein import into nucleus; nuclear localization sequence binding; protein transporter activity; nuclear membrane	Exportin-1/Importin-beta-like; Armadillo-type fold
**PVIT_0005329**	Phyca11_103899, PITG_01795T0, KUG00625, Phal07880, PMUR_0001267, PVIT_0005329	522	13.31	2.63E-04	1.65E-02	no	RNA binding	YTH domain
**PVIT_0014035**	Phyca11_18813, PITG_10990T0, KUF86772, Phal13704, PMUR_0002981, PVIT_0014035	663	13.22	2.77E-04	1.71E-02	no	dimethylallyltranstransferase activity; geranyltranstransferase activity; cytoplasm; isoprenoid biosynthetic process; integral component of membrane; farnesyl diphosphate biosynthetic process	Polyprenyl synthetase; Isoprenoid synthase domain superfamily
**PVIT_0013247**	Phyca11_509394, PITG_16466T0, KUF74988, Phal11912, PMUR_0009691, PVIT_0013247	624	13.17	2.84E-04	1.73E-02	no	methyltransferase activity; transferase activity; methylation	
**PVIT_0002890**	Phyca11_526931, PITG_10461T0, KUF98786, Phal09730, PMUR_0004401, PVIT_0002890	2895	13.03	3.07E-04	1.84E-02	no	protein folding in endoplasmic reticulum; ER membrane protein complex	ER membrane protein complex subunit 1, C-terminal; ER membrane protein complex subunit 1; Quinoprotein alcohol dehydrogenase-like superfamily; Pyrrolo-quinoline quinone repeat
**PVIT_0007430**	Phyca11_532846, PITG_02048T0, KUF89727, Phal06707, PMUR_0010425, PVIT_0007430	687	12.99	3.14E-04	1.85E-02	no	nucleus; tricarboxylic acid cycle; DNA replication; transferase activity; transferase activity, transferring acyl groups, acyl groups converted into alkyl on transfer	DNA polymerase subunit Cdc27
**PVIT_0014721**	Phyca11_511401, PITG_18434T0, KUF80971, Phal00587, PMUR_0009450, PVIT_0014721	573	12.91	3.27E-04	1.91E-02	no		B-block binding subunit of TFIIIC
**PVIT_0008239**	Phyca11_510870, PITG_06740T0, KUF88260, Phal13237, PMUR_0006086, PVIT_0008239	648	12.80	3.47E-04	1.99E-02	no	kinase activity; phosphorylation	PH-like domain superfamily
**PVIT_0001411**	Phyca11_548533, PITG_02952T0, KUF66944, Phal10413, PMUR_0008440, PVIT_0001411	1200	12.65	3.76E-04	2.10E-02	no	protein binding; kinase activity; phosphorylation	Tetratricopeptide repeat-containing domain; Tetratricopeptide-like helical domain superfamily; Tetratricopeptide repeat 1; Tetratricopeptide repeat

Tested gene	Orthogroup	Align. size	Delta LRT	*P* -value	Q-value	Secreted	GO terms	InterPro domains
**PVIT_0001830**	Phyca11_118786, PITG_00620T0, KUF76974, Phal03586, PMUR_0003899, PVIT_0001830	888	12.65	3.76E-04	2.10E-02	no	microtubule motor activity; binding; ATP binding; microtubule-based movement; microtubule binding; hydrolase activity	Kinesin motor domain; P-loop containing nucleoside triphosphate hydrolase
**PVIT_0004386**	Phyca11_560447, PITG_09437T0, KUF76756, Phal05034, PMUR_0007734, PVIT_0004386	306	12.49	4.10E-04	2.26E-02	no	deadenylation-dependent decapping of nuclear-transcribed mRNA; RNA cap binding; P-body; nucleus	LSM domain, eukaryotic/archaea-type; LSM domain superfamily
**PVIT_0013244**	Phyca11_511177, PITG_16468T0, KUF74967, Phal03634, PMUR_0009687, PVIT_0013244	1887	12.44	4.21E-04	2.29E-02	no	ATP binding; phosphorylation; inositol pentakisphosphate 2-kinase activity; intracellular part; single-organism process	Inositol-pentakisphosphate 2-kinase
**PVIT_0002656**	Phyca11_526539, PITG_06232T0, KUF83513, Phal11618, PMUR_0013156, PVIT_0002656	153	12.40	4.30E-04	2.31E-02	no		
**PVIT_0006578**	Phyca11_571248, PITG_11063T0, KUF82691, Phal07237, PMUR_0010212, PVIT_0006578	936	12.30	4.54E-04	2.40E-02	no	proteasome complex; protein binding; proteasome-mediated ubiquitin-dependent protein catabolic process	JAB1/MPN/MOV34 metalloenzyme domain; Rpn11/EIF3F, C-terminal
**PVIT_0012117**	Phyca11_573771, PITG_13918T0, KUF81328, Phal13689, PMUR_0006408, PVIT_0012117	2349	12.20	4.77E-04	2.49E-02	no	peptide alpha-N-acetyltransferase activity; protein binding; cytoplasm; N-terminal peptidyl-methionine acetylation	Tetratricopeptide repeat-containing domain; N-terminal acetyltransferase A, auxiliary subunit; Tetratricopeptide-like helical domain superfamily; Tetratricopeptide repeat 2; Tetratricopeptide repeat
**PVIT_0011519**	Phyca11_561723, PITG_04645T0, KUG00480, Phal08717, PMUR_0002719, PVIT_0011519	261	12.02	5.27E-04	2.71E-02	no	maintenance of transcriptional fidelity during DNA-templated transcription elongation from RNA polymerase II promoter; DNA-directed 5'-3' RNA polymerase activity; binding; DNA-directed RNA polymerase II, core complex; transcription-coupled nucleotide-excision repair; transcription initiation from RNA polymerase II promoter	DNA-directed RNA polymerase M, 15kDa subunit, conserved site; DNA-directed RNA polymerase, M/15kDa subunit
**PVIT_0002330**	Phyca11_536953, PITG_06358T0, KUG00093, Phal04000, PMUR_0006800, PVIT_0002330	6957	11.93	5.51E-04	2.80E-02	no	1,3-beta-D-glucan synthase complex; 1,3-beta-D-glucan synthase activity; (1->3)-beta-D-glucan biosynthetic process; membrane; integral component of membrane; transmembrane transporter activity; transmembrane transport	Major facilitator superfamily domain; 1,3-beta-glucan synthase subunit FKS1-like, domain-1; Glycosyl transferase, family 48; Major facilitator, sugar transporter-like
**PVIT_0017543**	Phyca11_105873, PITG_08806T0, KUF88912, Phal00085, PMUR_0009543, PVIT_0017543	2775	11.86	5.75E-04	2.89E-02	no	calcium-dependent cysteine-type endopeptidase activity; intracellular; cytoplasm; proteolysis	Peptidase C2, calpain, catalytic domain; Peptidase C2, calpain, large subunit, domain III; Peptidase C2, calpain, domain III; Peptidase C2, calpain family
**PVIT_0003723**	Phyca11_6928, PITG_15920T0, KUF84589, Phal09845, PMUR_0000189, PVIT_0003723	693	11.68	6.33E-04	3.06E-02	no	integral component of membrane	Transmembrane protein TMEM64
**PVIT_0005563**	Phyca11_35790, PITG_07145T0, KUF77196, Phal04042, PMUR_0000822, PVIT_0005563	975	11.70	6.25E-04	3.06E-02	no	calcium ion binding; peroxisome; fatty acid biosynthetic process; malonyl-CoA decarboxylase activity	Malonyl-CoA decarboxylase, C-terminal
**PVIT_0012806**	Phyca11_525423, PITG_19851T0, KUF82130, Phal12053, PMUR_0011389, PVIT_0012806	1653	11.69	6.30E-04	3.06E-02	no	DNA binding	SANT/Myb domain; Myb domain; Homeobox-like domain superfamily
**PVIT_0023713**	Phyca11_109643, PITG_05359T0, KUG00654, Phal08368, PMUR_0010774, PVIT_0023713	2934	11.56	6.74E-04	3.22E-02	no	binding	Telomere-associated protein Rif1, N-terminal; Armadillo-type fold
**PVIT_0025164**	Phyca11_16573, PITG_13434T0, KUG01795, Phal08065, PMUR_0006374, PVIT_0025164	1539	11.53	6.83E-04	3.23E-02	yes	protein binding; integral component of membrane	Tetratricopeptide-like helical domain superfamily; Sel1-like repeat
**PVIT_0008790**	Phyca11_507044, PITG_06514T0, KUF88950, Phal01618, PMUR_0009358, PVIT_0008790	447	11.02	9.02E-04	4.21E-02	no	DNA binding; DNA-directed 5'-3' RNA polymerase activity; calcium ion binding	EF-Hand 1, calcium-binding site; EF-hand domain; RNA polymerase, beta subunit, protrusion; EF-hand domain pair
**PVIT_0010265**	Phyca11_510199, PITG_17157T0, KUF64291, Phal14163, PMUR_0005615, PVIT_0010265	762	10.91	9.57E-04	4.42E-02	no	nucleotide binding; nucleic acid binding	RNA recognition motif domain; Nucleotide-binding alpha-beta plait domain superfamily

Tested gene	Orthogroup	Align. size	Delta LRT	*P*-value	Q-value	Secreted	GO terms	InterPro domains
**PVIT_0015981**	Phyca11_551179, PITG_04917T0, KUF80394, Phal14097, PMUR_0007136, PVIT_0015981	1530	10.83	9.99E-04	4.56E-02	no	protein binding; anaphase-promoting complex; regulation of mitotic metaphase/anaphase transition	Cdc23; Tetratricopeptide repeat-containing domain; Tetratricopeptide-like helical domain superfamily; Tetratricopeptide repeat 1; Tetratricopeptide repeat
**PVIT_0001226**	Phyca11_506190, PITG_02698T0, KUF84935, Phal08820, PMUR_0001030, PVIT_0001226	534	10.77	1.03E-03	4.61E-02	no	binding	Armadillo-type fold
**PVIT_0008576**	Phyca11_525308, PITG_06889T0, KUF91944, Phal11045, PMUR_0007434, PVIT_0008576	1887	10.77	1.03E-03	4.61E-02	no	proteolysis; exocytosis; integral component of membrane; dipeptidase activity	Peptidase C69, dipeptidase A
**PVIT_0000445**	Phyca11_548193, PITG_03266T0, KUF69650, Phal04494, PMUR_0004883, PVIT_0000445	600	10.52	1.18E-03	5.05E-02	no		
**PVIT_0000656**	Phyca11_534689, PITG_03458T0, KUG02205, Phal03704, PMUR_0000261, PVIT_0000656	840	10.50	1.19E-03	5.05E-02	no		Pleckstrin homology domain; ELMO domain; PH-like domain superfamily
**PVIT_0007171**	Phyca11_548255, PITG_17258T0, KUF77216, Phal14089, PMUR_0007037, PVIT_0007171	1164	10.57	1.15E-03	5.05E-02	no	GTP binding	GTP binding domain; Uncharacterised GTP-binding protein, C-terminal; P-loop containing nucleoside triphosphate hydrolase
**PVIT_0007431**	Phyca11_562149, PITG_02047T0, KUF89726, Phal06708, PMUR_0010426, PVIT_0007431	912	10.48	1.21E-03	5.05E-02	no	epithelial cilium movement; DNA helicase activity; ATP binding; nucleus; DNA repair; DNA recombination; transcription, DNA-templated; heart development; DNA duplex unwinding; ATP-dependent 5'-3' DNA helicase activity; motile cilium assembly; negative regulation of transcription, DNA-templated; digestive tract development; regulation of heart growth; axonemal dynein complex assembly	AAA+ ATPase domain; TIP49, C-terminal; RuvB-like; Nucleic acid-binding, OB-fold; P-loop containing nucleoside triphosphate hydrolase
**PVIT_0008480**	Phyca11_503137, PITG_06796T0, KUF86440, Phal05304, PMUR_0006526, PVIT_0008480	798	10.49	1.20E-03	5.05E-02	no	protein kinase activity; ATP binding; protein phosphorylation	Protein kinase domain; Protein kinase-like domain superfamily
**PVIT_0010560**	Phyca11_572114, PITG_09827T0, KUF80614, Phal00324, PMUR_0000416, PVIT_0010560	705	10.52	1.18E-03	5.05E-02	no	kinetochore; protein binding; kinetochore microtubule; mitotic spindle assembly checkpoint; phragmoplast; mitotic checkpoint complex; ubiquitin binding; bub1-bub3 complex	WD40-repeat-containing domain; WD40/YVTN repeat-like-containing domain superfamily; WD40 repeat
**PVIT_0012401**	Phyca11_18432, PITG_04027T0, KUG01718, Phal08460, PMUR_0007470, PVIT_0012401	1299	10.44	1.24E-03	5.12E-02	no	integral component of membrane	
**PVIT_0004375**	Phyca11_503853, PITG_09432T0, KUF76754, Phal05064, PMUR_0007741, PVIT_0004375	1440	10.31	1.32E-03	5.32E-02	no	rRNA modification; protein binding; snoRNA binding; box C/D snoRNP complex; small-subunit processome	Nop domain; NOP5, N-terminal; NOSIC
**PVIT_0015139**	Phyca11_41439, PITG_11813T0, KUF76193, Phal08159, PMUR_0004175, PVIT_0015139	462	10.30	1.33E-03	5.32E-02	no	single-organism process	KIF-1 binding protein
**PVIT_0018513**	Phyca11_508797, PITG_12489T0, KUF64475, Phal08555, PMUR_0009815, PVIT_0018513	1797	10.29	1.34E-03	5.32E-02	no	nucleus; cytosol; regulation of transcription, DNA-templated; protein methylation; methyltransferase activity; histone-arginine N-methyltransferase activity; peptidyl-arginine methylation, to symmetrical-dimethyl arginine; histone arginine methylation; peptidyl-arginine N-methylation	Protein arginine N-methyltransferase PRMT5; Protein arginine N-methyltransferase
**PVIT_0021050**	Phyca11_558170, PITG_13014T0, KUF76541, Phal03980, PMUR_0006766, PVIT_0021050	1161	10.31	1.33E-03	5.32E-02	no	DNA binding; aminopeptidase activity; proteolysis; metallopeptidase activity	Peptidase M24; ArsR-like helix-turn-helix domain; Peptidase M24, methionine aminopeptidase; PA2G4 family
**PVIT_0003374**	Phyca11_39056, PITG_05796T0, KUF93703, Phal08266, PMUR_0003949, PVIT_0003374	681	10.24	1.37E-03	5.41E-02	no	helicase activity; nucleolus; methyltransferase activity; S-adenosylmethionine-dependent methyltransferase activity; methylation	Ribosomal RNA processing protein 8
**PVIT_0012102**	Phyca11_530140, PITG_13901T0, KUF87461, Phal10533, PMUR_0006423, PVIT_0012102	2223	10.20	1.41E-03	5.48E-02	no	protein binding	Leucine-rich repeat

Tested gene	Orthogroup	Align. size	Delta LRT	*P* -value	Q-value	Secreted	GO terms	InterPro domains
**PVIT_0003624**	Phyca11_510275, PITG_17786T0, KUF78419, Phal14118, PMUR_0005879, PVIT_0003624	1311	10.11	1.48E-03	5.71E-02	no	catalytic activity; argininosuccinate lyase activity; cytosol; arginine biosynthetic process via ornithine; protein tetramerization	Fumarate lyase, conserved site; Fumarate lyase, N-terminal; Fumarate lyase family; Argininosuccinate lyase; L-Aspartase-like; Fumarase/histidase, N-terminal
**PVIT_0000959**	Phyca11_117156, PITG_02419T0, KUF81990, Phal03620, PMUR_0003474, PVIT_0000959	1134	10.03	1.54E-03	5.90E-02	no	ion channel activity; voltage-gated ion channel activity; voltage-gated potassium channel activity; transport; ion transport; potassium ion transport; voltage-gated potassium channel complex; membrane; integral component of membrane; ion transmembrane transport; regulation of ion transmembrane transport; transmembrane transport; potassium ion transmembrane transport	Ion transport domain; Potassium channel domain
**PVIT_0003069**	Phyca11_106683, PITG_10252T0, KUG00411, Phal14188, PMUR_0005817, PVIT_0003069	414	10.00	1.57E-03	5.94E-02	no	nucleic acid binding	
**PVIT_0000873**	Phyca11_538407, PITG_02256T0, KUF88613, Phal01432, PMUR_0004466, PVIT_0000873	1194	9.93	1.63E-03	6.09E-02	no	catalytic activity; L-aspartate:2-oxoglutarate aminotransferase activity; mitochondrion; cellular amino acid metabolic process; transaminase activity; biosynthetic process; pyridoxal phosphate binding; identical protein binding; L-phenylalanine:2-oxoglutarate aminotransferase activity	Aminotransferases, class-I, pyridoxal-phosphate-binding site; Aminotransferase, class I/classII; Aspartate/other aminotransferase; Pyridoxal phosphate-dependent transferase, major domain; Pyridoxal phosphate-dependent transferase
**PVIT_0001204**	Phyca11_545592, PITG_02636T0, KUF88357, Phal07595, PMUR_0001246, PVIT_0001204	636	9.92	1.64E-03	6.09E-02	no	peroxisome	Pex19 protein
**PVIT_0016184**	Phyca11_5519, PITG_15748T0, KUF73660, Phal08511, PMUR_0002609, PVIT_0016184	837	9.85	1.70E-03	6.25E-02	no	protein binding; isomerase activity	Tetratricopeptide repeat-containing domain; Peptidyl-prolyl cis-trans isomerase, FKBP-type; Tetratricopeptide-like helical domain superfamily; Tetratricopeptide repeat
**PVIT_0004577**	Phyca11_34812, PITG_09792T0, KUF80477, Phal07279, PMUR_0007242, PVIT_0004577	1200	9.73	1.81E-03	6.62E-02	no	nucleic acid binding; ATP-dependent RNA helicase activity; ATP binding; RNA secondary structure unwinding; ion binding	Helicase, C-terminal; DEAD/DEAH box helicase domain; Helicase superfamily 1/2, ATP-binding domain; RNA helicase, DEAD-box type, Q motif; P-loop containing nucleoside triphosphate hydrolase
**PVIT_0006926**	Phyca11_571672, PITG_12226T0, KUF86600, Phal01085, PMUR_0008751, PVIT_0006926	7878	9.66	1.88E-03	6.80E-02	no	protein serine/threonine kinase activity; binding; protein binding; ATP binding; nucleus; DNA repair; protein phosphorylation; integral component of membrane; kinase activity; macromolecular complex binding	Phosphatidylinositol 3/4-kinase, conserved site; Phosphatidylinositol 3-/4-kinase, catalytic domain; PIK-related kinase, FAT; FATC domain; FKBP12-rapamycin binding domain; PIK-related kinase; Domain of unknown function DUF3385, target of rapamycin protein; Serine/threonine-protein kinase TOR; Protein kinase-like domain superfamily; Armadillo-like helical; Tetratricopeptide-like helical domain superfamily; Armadillo-type fold
**PVIT_0008172**	Phyca11_511439, PITG_09282T0, KUG00452, Phal10033, PMUR_0003742, PVIT_0008172	3102	9.61	1.93E-03	6.93E-02	no	nucleotide binding; ATP binding; endoplasmic reticulum; integral component of plasma membrane; integral component of membrane; cation-transporting ATPase activity; metal ion binding; cation transmembrane transport	Cation-transporting P-type ATPase, N-terminal; Cation-transporting P-type ATPase, C-terminal; P-type ATPase; P-type ATPase, A domain superfamily; HAD superfamily; P-type ATPase, transmembrane domain superfamily; P-type ATPase, cytoplasmic domain N; P-type ATPase, phosphorylation site
**PVIT_0015709**	Phyca11_530603, PITG_20263T0, KUF87812, Phal10051, PMUR_0003160, PVIT_0015709	477	9.50	2.05E-03	7.29E-02	no		
**PVIT_0003669**	Phyca11_505826, PITG_15847T0, KUF80597, Phal00683, PMUR_0004281, PVIT_0003669	324	9.44	2.12E-03	7.41E-02	no	integral component of membrane	
**PVIT_0006721**	Phyca11_504657, PITG_11215T0, KUF68290, Phal10588, PMUR_0009239, PVIT_0006721	1239	9.45	2.12E-03	7.41E-02	no	DNA binding; transcription factor activity, sequence-specific DNA binding; protein binding; nucleus; regulation of transcription, DNA-templated; membrane; integral component of membrane; sequence-specific DNA binding	DnaJ domain, conserved site; DnaJ domain; Tetratricopeptide repeat-containing domain; Tetratricopeptide-like helical domain superfamily; Tetratricopeptide repeat 1; Tetratricopeptide repeat 2; Tetratricopeptide repeat

Tested gene	Orthogroup	Align. size	Delta LRT	*P*-value	Q-value	Secreted	GO terms	InterPro domains
**PVIT_0014447**	Phyca11_127143, PITG_16078T0, KUF92384, Phal13077, PMUR_0005325, PVIT_0014447	3390	9.42	2.14E-03	7.43E-02	no	protein binding; ligase activity	Tetratricopeptide-like helical domain superfamily; Pentatricopeptide repeat
**PVIT_0019916**	Phyca11_507990, PITG_10675T0, KUF86043, Phal09097, PMUR_0008814, PVIT_0019916	681	9.39	2.18E-03	7.47E-02	no	protein kinase activity; protein serine/threonine kinase activity; ATP binding; protein phosphorylation; integral component of membrane	Serine/threonine-protein kinase, active site; Protein kinase, ATP binding site; Protein kinase domain; Protein kinase-like domain superfamily
PVIT_0009280	Phyca11_532120, PITG_03746T0, KUF64635, Phal01178, PMUR_0003371, PVIT_0009280	702	9.34	2.25E-03	7.64E-02	no	phosphatidylinositol binding	Phox homologous domain
**PVIT_0004430**	Phyca11_5246, PITG_09508T0, KUF87566, Phal04971, PMUR_0007503, PVIT_0004430	903	9.29	2.31E-03	7.72E-02	no	thiol-dependent ubiquitin-specific protease activity; protein binding; intracellular; nucleus; cytoplasm; ubiquitin-dependent protein catabolic process; shoot system morphogenesis; protein deubiquitination; leaf development	Peptidase C12, ubiquitin carboxyl-terminal hydrolase; Ubiquitinyl hydrolase, UCH37 type
**PVIT_0022467**	Phyca11_531502, PITG_12871T0, KUF85714, Phal07421, PMUR_0011340, PVIT_0022467	1659	9.30	2.29E-03	7.72E-02	no	maturation of SSU-rRNA from tricistronic rRNA transcript (SSU-rRNA, 5.8S rRNA, LSU-rRNA); protein binding; nucleolus; snoRNA binding; 90S preribosome; RENT complex; small-subunit processome; rDNA heterochromatin; t-UTP complex; positive regulation of transcription from RNA polymerase I promoter	WD40 repeat, conserved site; WD40-repeat-containing domain; Quinoprotein alcohol dehydrogenase-like superfamily; WD40/YVTN repeat-like-containing domain superfamily; WD40 repeat
**PVIT_0006958**	Phyca11_509288, PITG_12191T0, KUG02054, Phal05048, PMUR_0005127, PVIT_0006958	1557	9.27	2.33E-03	7.75E-02	no		
**PVIT_0006664**	Phyca11_504624, PITG_11171T0, KUG00077, Phal11472, PMUR_0003607, PVIT_0006664	1305	9.14	2.51E-03	8.25E-02	no	endosome; endocytosis; vesicle organization; extrinsic component of membrane; phosphatidylinositol binding	Phox homologous domain; AH/BAR domain superfamily
**PVIT_0019187**	Phyca11_112787, PITG_08708T0, KUG02168, Phal02063, PMUR_0010310, PVIT_0019187	1752	9.08	2.58E-03	8.42E-02	no	nucleotide binding; nucleic acid binding; RNA binding; transferase activity, transferring glycosyl groups	RNA recognition motif domain; RNA recognition motif domain, eukaryote; Nucleotide-binding alpha-beta plait domain superfamily
**PVIT_0018337**	Phyca11_528327, PITG_21219T0, KUF84842, Phal07762, PMUR_0008572, PVIT_0018337	1206	9.02	2.67E-03	8.59E-02	no	nucleotide binding; nucleic acid binding; DNA binding; nucleus; transcription, DNA-templated; regulation of transcription, DNA-templated	RNA recognition motif domain; UPF3 domain; Nucleotide-binding alpha-beta plait domain superfamily
**PVIT_0022458**	Phyca11_502634, PITG_12837T0, KUF82068, Phal07425, PMUR_0011413, PVIT_0022458	762	9.02	2.67E-03	8.59E-02	no	nitrogen compound metabolic process; hydrolase activity, acting on carbon-nitrogen (but not peptide) bonds	Carbon-nitrogen hydrolase
**PVIT_0021053**	Phyca11_538207, PITG_13017T0, KUF76543, Phal03983, PMUR_0006763, PVIT_0021053	3054	8.95	2.78E-03	8.86E-02	no	nucleotide binding; nucleic acid binding; ATP-dependent RNA helicase activity; helicase activity; ATP binding; nucleus; cytoplasm; RNA processing; ATP-dependent helicase activity; transferase activity	DNA/RNA helicase, ATP-dependent, DEAH-box type, conserved site; Helicase, C-terminal; AAA+ ATPase domain; Helicase-associated domain; DEAD/DEAH box helicase domain; Domain of unknown function DUF1605; Helicase superfamily 1/2, ATP-binding domain; P-loop containing nucleoside triphosphate hydrolase
**PVIT_0011532**	Phyca11_526141, PITG_04651T0, KUF81026, Phal03557, PMUR_0002704, PVIT_0011532	3273	8.90	2.86E-03	9.04E-02	no	nuclear export signal receptor activity; binding; nuclear pore; cytoplasm; protein export from nucleus; intracellular protein transport; Ran GTPase binding	Importin-beta, N-terminal domain; Armadillo-like helical; Armadillo-type fold
**PVIT_0020049**	Phyca11_502825, PITG_02203T0, KUF76695, Phal00507, PMUR_0003517, PVIT_0020049	1713	8.87	2.90E-03	9.11E-02	no	metal ion binding	FYVE zinc finger; Zinc finger, FYVE-related; Zinc finger, FYVE/PHD-type; Zinc finger, RING/FYVE/PHD-type
**PVIT_0010005**	Phyca11_532048, PITG_19361T0, KUF78680, Phal08207, PMUR_0005191, PVIT_0010005	1095	8.80	3.01E-03	9.37E-02	no	DNA binding; nucleus; transcription, DNA-templated; regulation of transcription, DNA-templated	SANT/Myb domain; Myb domain, plants; Myb domain; Homeobox-like domain superfamily

Tested gene	Orthogroup	Align. size	Delta LRT	*P* -value	Q-value	Secreted	GO terms	InterPro domains
**PVIT_0002851**	Phyca11_564360, PITG_10525T0, KUF97566, Phal00241, PMUR_0008019, PVIT_0002851	687	8.76	3.08E-03	9.46E-02	no	nucleic acid binding; metal ion binding	Zinc finger C2H2-type
**PVIT_0013001**	Phyca11_7431, PITG_01363T0, KUF97689, Phal01606, PMUR_0001089, PVIT_0013001	1236	8.76	3.08E-03	9.46E-02	no		

Orthogroup: orthology group used for the branch-site test. Align. size: size of the alignment for the computation of likelihoods. Delta LRT: test statistic value for the likelihood ratio test. *P* -values were computed considering that the test statistic followed a chi-square distribution with 1 degree of freedom. *Q* -values were computed using a FDR procedure. Genes found expressed in the RNA-seq data are in bold.

Supplementary table 14 *Pl. muralis* genes with a significant signal in the branch-site test

Tested gene	Orthogroup	Align. size	Delta LRT	*P*-value	*Q*-value	Secreted	GO terms	InterPro domains
PMUR_0001567	Phyca11_527201, PITG_05560T0, KUF76730, Phal07792, PMUR_0001567, PVIT_0006127	1773	142.22	8.71E-33	3.50E-29	no		Transmembrane protein TqsA-like
PMUR_0003310	Phyca11_575261, PITG_16801T0, KUF76457, Phal02727, PMUR_0003310, PVIT_0005623	2427	140.05	2.60E-32	5.22E-29	no	protein binding; nucleus; transcription, DNA-templated; regulation of transcription, DNA-templated	WD40 repeat, conserved site; TUP1-like enhancer of split; WD40-repeat-containing domain; WD40/YVTN repeat-like-containing domain superfamily; WD40 repeat; G-protein beta WD-40 repeat
PMUR_0003488	Phyca11_569416, PITG_02432T0, KUF82000, Phal09734, PMUR_0003488, PVIT_0000975	1557	133.54	6.90E-31	9.24E-28	no	endoplasmic reticulum; gamma-tubulin ring complex; integral component of membrane; transferase activity, transferring hexosyl groups; gamma-tubulin complex localization	Glycosyltransferase, ALG3; Diphthamide synthesis DPH1/DPH2; Mitotic-spindle organizing protein 1
PMUR_0007058	Phyca11_534782, PITG_17298T0, KUF77837, Phal11291, PMUR_0007058, PVIT_0007143	1797	131.60	1.83E-30	1.84E-27	no	nucleic acid binding; RNA binding; methyltransferase activity; zinc ion binding; tRNA (cytosine-5-)-methyltransferase activity; tRNA methylation	Bacterial Fmu (Sun)/eukaryotic nucleolar NOL1/Nop2p, conserved site; SAM-dependent methyltransferase RsmB/NOP2-type; Zinc finger, CCHC-type; RNA (C5-cytosine) methyltransferase
PMUR_0001117	Phyca11_506369, PITG_01402T0, KUG01891, Phal05882, PMUR_0001117, PVIT_0013032	960	127.97	1.14E-29	9.15E-27	no	protein binding; metal ion binding	Ankyrin repeat-containing domain; Ankyrin repeat
PMUR_0004400	Phyca11_73678, PITG_10460T0, KUF98791, Phal08974, PMUR_0004400, PVIT_0002889	4497	108.06	2.61E-25	1.75E-22	no	metal ion binding	Zinc finger, CCCH-type; P-loop containing nucleoside triphosphate hydrolase
PMUR_0005013	Phyca11_562024, PITG_01941T0, KUF91124, Phal05002, PMUR_0005013, PVIT_0013721	1401	104.51	1.57E-24	8.99E-22	no	DNA binding	SANT/Myb domain; Myb-like domain; Myb domain; Homeobox-like domain superfamily; Leucine-rich repeat, cysteine-containing subtype
PMUR_0007681	Phyca11_102611, PITG_15241T0, KUG01569, Phal02187, PMUR_0007681, PVIT_0009132	1506	99.92	1.59E-23	7.96E-21	yes		EGF-like, conserved site; EGF-like domain; EGF-like domain, extracellular
PMUR_0001531	Phyca11_117367, PITG_01173T0, KUF79081, Phal08698, PMUR_0001531, PVIT_0010408	600	95.03	1.87E-22	8.36E-20	no	transferase activity	
PMUR_0002275	Phyca11_112853, PITG_07307T0, KUF73082, Phal02050, PMUR_0002275, PVIT_0006848	5376	81.50	1.76E-19	7.06E-17	yes	protein binding	PA14 domain; Galactose-binding-like domain superfamily; Immunoglobulin-like fold; Immunoglobulin E-set; Filamin/ABP280 repeat; Filamin/ABP280 repeat-like
PMUR_0007021	Phyca11_114386, PITG_18010T0, KUF85090, Phal03365, PMUR_0007021, PVIT_0017815	951	74.68	5.53E-18	2.02E-15	no	nucleus	XAP5 protein
PMUR_0001949	Phyca11_117803, PITG_04001T0, KUF77694, Phal06371, PMUR_0001949, PVIT_0017233	918	70.18	5.40E-17	1.81E-14	no	cysteine-type endopeptidase activity; metalloendopeptidase activity; membrane; integral component of endoplasmic reticulum membrane; CAAX-box protein processing	CAAX amino terminal protease
PMUR_0006066	Phyca11_5774, PITG_06715T0, KUF88877, Phal14365, PMUR_0006066, PVIT_0008218	891	68.03	1.61E-16	4.98E-14	no	phosphoprotein phosphatase activity; protein dephosphorylation; hydrolase activity	Calcineurin-like phosphoesterase domain, ApaH type; Serine/threonine-specific protein phosphatase/bis(5-nucleosyl)-tetraphosphatase
PMUR_0002613	Phyca11_5513, PITG_15779T0, KUG00870, Phal08503, PMUR_0002613, PVIT_0016188	1308	67.78	1.83E-16	5.25E-14	no	RNA binding; calcium ion binding	EF-Hand 1, calcium-binding site; DnaJ domain; EF-hand domain; cAMP-dependent protein kinase regulatory subunit, dimerization-anchoring domain; K Homology domain, type 1; EF-hand domain pair
PMUR_0007729	Phyca11_525738, PITG_09447T0, KUF76766, Phal05022, PMUR_0007729, PVIT_0004392	3705	67.24	2.41E-16	6.44E-14	no	peptide alpha-N-acetyltransferase activity; protein binding; N-terminal peptidyl-methionine acetylation; NatB complex	WD40 repeat, conserved site; WD40-repeat-containing domain; Fyv7/TAP26; N-acetyltransferase B complex, non-catalytic subunit; Tetratricopeptide-like helical domain superfamily; WD40/YVTN repeat-like-containing domain superfamily; WD40 repeat
PMUR_0005277	Phyca11_508083, PITG_02503T0, KUF82417, Phal02332, PMUR_0005277, PVIT_0001050	846	64.99	7.53E-16	1.89E-13	no	catalytic activity; metabolic process	Phosphoadenosine phosphosulphate reductase; Rossmann-like alpha/beta/alpha sandwich fold
PMUR_0011578	Phyca11_507107, PITG_06631T0, KUF85240, Phal00047, PMUR_0011578, PVIT_0008718	1278	63.85	1.34E-15	3.17E-13	no	integral component of membrane; phosphatidylinositol binding	Phox homologous domain; Choline transporter-like
PMUR_0004875	Phyca11_115006, PITG_03283T0, KUF69644, Phal01385, PMUR_0004875, PVIT_0000453	1197	56.71	5.05E-14	1.13E-11	yes	thiol-dependent ubiquitin-specific protease activity; protein binding; proteolysis	F-box domain; Peptidase C19, ubiquitin-specific peptidase, DUSP domain
PMUR_0001980	Phyca11_528810, PITG_03943T0, KUF90630, Phal13935, PMUR_0001980, PVIT_0017200	321	54.22	1.79E-13	3.78E-11	no		
PMUR_0010325	Phyca11_573197, PITG_07841T0, KUF80120, Phal05566, PMUR_0010325, PVIT_0010839	1431	54.09	1.91E-13	3.84E-11	no	nucleotide binding; aminoacyl-tRNA ligase activity; glycine-tRNA ligase activity; ATP binding; cytoplasm; tRNA aminoacylation for protein translation; glycyl-tRNA aminoacylation	Aminoacyl-tRNA synthetase, class II (G/ P/ S/T); Anticodon-binding; Aminoacyl-tRNA synthetase, class II; Glycyl-tRNA synthetase; Glycyl-tRNA synthetase/DNA polymerase subunit gamma-2

Tested gene	Orthogroup	Align. size	Delta LRT	*P*-value	*Q*-value	Secreted	GO terms	InterPro domains
PMUR_0004180	Phyca11_41303, PITG_11805T0, KUF79758, Phal02166, PMUR_0004180, PVIT_0015144	450	51.02	9.13E-13	1.75E-10	no	nucleotide binding; nucleic acid binding	RNA recognition motif domain
PMUR_0007200	Phyca11_99433, PITG_06944T0, KUF90117, Phal08541, PMUR_0007200, PVIT_0018116	189	45.06	1.91E-11	3.49E-09	no		
PMUR_0002593	Phyca11_504150, PITG_15696T0, KUF86113, Phal09989, PMUR_0002593, PVIT_0016169	2031	43.84	3.56E-11	6.23E-09	no	nucleic acid binding; DNA binding; ATP-dependent DNA helicase activity; ATP binding; nucleus; nucleobase-containing compound metabolic process; nucleotide-excision repair; ATP-dependent helicase activity; DNA duplex unwinding	DNA/RNA helicase, ATP-dependent, DEAH-box type, conserved site; Helicase-like, DEXD box c2 type; ATP-dependent helicase, C-terminal; DEAD2; Helical and beta-bridge domain; Helicase superfamily 1/2, ATP-binding domain, DinG/Rad3-type; RAD3/XPD family; ATP-dependent helicase Rad3/Chl1-like; P-loop containing nucleoside triphosphate hydrolase
PMUR_0004879	Phyca11_507482, PITG_03275T0, KUF69635, Phal04866, PMUR_0004879, PVIT_0000449	936	41.95	9.36E-11	1.55E-08	no	ATP binding; nucleus; microtubule-severing ATPase activity; cytoplasmic microtubule organization	ATPase, AAA-type, conserved site; ATPase, AAA-type, core; MIT; Vps4 oligomerisation, C-terminal; P-loop containing nucleoside triphosphate hydrolase
PMUR_0005294	Phyca11_126376, PITG_16118T0, KUF79594, Phal13797, PMUR_0005294, PVIT_0014415	876	41.89	9.63E-11	1.55E-08	no	metal ion binding	WIBG, Mago-binding; Alpha-ketoglutarate-dependent dioxygenase AlkB-like
PMUR_0009520	Phyca11_505136, PITG_08834T0, KUF98811, Phal04384, PMUR_0009520, PVIT_0017554	219	39.60	3.11E-10	4.81E-08	no	RNA binding; ribosome	
PMUR_0002542	Phyca11_11834, PITG_18455T0, KUG01019, Phal06479, PMUR_0002542, PVIT_0014700	375	39.46	3.34E-10	4.98E-08	no		Complex 1 LYR protein
PMUR_0009897	Phyca11_6670, PITG_17028T0, KUF83453, Phal14352, PMUR_0009897, PVIT_0008810	6555	38.48	5.52E-10	7.92E-08	no	protein binding	cDENN domain; Tetratricopeptide-like helical domain superfamily
PMUR_0001897	Phyca11_84261, PITG_00939T0, KUF78034, Phal02269, PMUR_0001897, PVIT_0007258	672	38.18	6.46E-10	8.95E-08	no		Diphthamide synthase domain; Endoribonuclease L-PSP/chorismate mutase-like; YjgF/YER057c/UK114 family; Rossmann-like alpha/beta/alpha sandwich fold
PMUR_0000837	Phyca11_20153, PITG_01525T0, KUF77185, Phal10401, PMUR_0000837, PVIT_0005547	537	37.92	7.38E-10	9.88E-08	no		FAM92 protein
PMUR_0005372	Phyca11_537973, PITG_14622T0, KUF79070, Phal02365, PMUR_0005372, PVIT_0016023	630	37.47	9.28E-10	1.20E-07	no	nucleus; regulation of exit from mitosis	
PMUR_0001680	Phyca11_4263, PITG_12984T0, KUF91955, Phal07113, PMUR_0001680, PVIT_0006460	897	34.54	4.18E-09	5.25E-07	no	integral component of membrane	Tetraspanin; Tetraspanin/Peripherin
PMUR_0010436	Phyca11_532851, PITG_02055T0, KUF89733, Phal09569, PMUR_0010436, PVIT_0007443	1092	33.94	5.67E-09	6.90E-07	no	protein binding; protein folding; prefoldin complex; unfolded protein binding	Tetratricopeptide repeat-containing domain; Prefoldin alpha-like; Tetratricopeptide-like helical domain superfamily; Tetratricopeptide repeat 2; Tetratricopeptide repeat
PMUR_0006374	Phyca11_16573, PITG_13434T0, KUG01795, Phal08065, PMUR_0006374, PVIT_0025164	1539	33.82	6.03E-09	7.13E-07	no	integral component of membrane	Sel1-like repeat
PMUR_0009118	Phyca11_552955, PITG_11907T0, KUF78523, Phal10251, PMUR_0009118, PVIT_0005967	465	33.41	7.47E-09	8.57E-07	no	diphthine synthase activity; metabolic process; methyltransferase activity; peptidyl-diphthamide biosynthetic process from peptidyl-histidine; methylation	Tetrapyrrole methylase; Diphthine synthase; Tetrapyrrole methylase, subdomain 2; Tetrapyrrole methylase, subdomain 1
PMUR_0006492	Phyca11_531357, PITG_18912T0, KUF84612, Phal13873, PMUR_0006492, PVIT_0017064	1797	33.25	8.08E-09	9.02E-07	no	nuclear-transcribed mRNA catabolic process, nonsense-mediated decay; DNA binding; helicase activity; ATP binding; cytoplasm; zinc ion binding	RNA helicase UPF1, UPF2-interacting domain; P-loop containing nucleoside triphosphate hydrolase
PMUR_0008455	Phyca11_548492, PITG_02924T0, KUF77925, Phal13246, PMUR_0008455, PVIT_0001441	1386	33.03	9.06E-09	9.83E-07	no		Pentatricopeptide repeat
PMUR_0004112	Phyca11_118922, PITG_00748T0, KUF96721, Phal12777, PMUR_0004112, PVIT_0001940	747	32.95	9.44E-09	9.97E-07	no	maturation of SSU-rRNA from tricistronic rRNA transcript (SSU-rRNA, 5.8S rRNA, LSU-rRNA); binding; transport; integral component of membrane; snoRNA binding; 90S preribosome; small-subunit processome; t-UTP complex; positive regulation of transcription from RNA polymerase I promoter	BP28, C-terminal domain; Major facilitator superfamily domain; Folate-biopterin transporter; U3 small nucleolar RNA-associated protein 10; Armadillo-like helical; Armadillo-type fold
PMUR_0005271	Phyca11_508077, PITG_02526T0, KUF76525, Phal05602, PMUR_0005271, PVIT_0001060	3987	30.96	2.63E-08	2.71E-06	no	microtubule motor activity; ATP binding; microtubule-based movement; ATPase activity; dynein complex	Sigma-54 interaction domain, ATP-binding site 1; AAA+ ATPase domain; Dynein heavy chain domain; ATPase, dynein-related, AAA domain; Dynein heavy chain, domain-1; Dynein heavy chain, domain-2; Dynein heavy chain, AAA module D4; Dynein heavy chain, coiled coil stalk; Dynein heavy chain; P-loop containing nucleoside triphosphate hydrolase

Tested gene	Orthogroup	Align. size	Delta LRT	P-value	Q-value	Secreted	GO terms	InterPro domains
PMUR_0013122	Phyca11_8928, PITG_02482T0, KUF89910, Phal04643, PMUR_0013122, PVIT_0001024	2658	30.71	3.00E-08	3.01E-06	no	mRNA splicing, via spliceosome; ATP-dependent RNA helicase activity; helicase activity; ATP binding; spliceosomal complex; cytoplasm	DNA/RNA helicase, ATP-dependent, DEAH-box type, conserved site; Helicase, C-terminal; AAA+ ATPase domain; Helicase-associated domain; Domain of unknown function DUF1605; Helicase superfamily 1/2, ATP-binding domain; P-loop containing nucleoside triphosphate hydrolase
PMUR_0002272	Phyca11_7912, PITG_07319T0, KUF76263, Phal14400, PMUR_0002272, PVIT_0006841	2721	30.59	3.18E-08	3.12E-06	no	GTPase activity; ion channel activity; voltage-gated potassium channel activity; GTP binding; mitochondrial inner membrane; ion transport; potassium ion transport; voltage-gated potassium channel complex; membrane; ribosome biogenesis; transmembrane transport; large conductance calcium-activated potassium channel activity; potassium ion transmembrane transport	Ion transport domain; GTP binding domain; Potassium channel, BK, alpha subunit; GTP-binding protein, orthogonal bundle domain superfamily; P-loop containing nucleoside triphosphate hydrolase
PMUR_0004578	Phyca11_552288, PITG_14171T0, KUF75707, Phal03709, PMUR_0004578, PVIT_0015222	1461	29.16	6.65E-08	6.36E-06	no	amino acid transmembrane transport; integral component of plasma membrane; amino acid transmembrane transporter activity; L-amino acid transmembrane transporter activity; antiporter activity; membrane; L-alpha-amino acid transmembrane transport	Amino acid/polyamine transporter I
PMUR_0002160	Phyca11_529849, PITG_01031T0, KUF89154, Phal07024, PMUR_0002160, PVIT_0009498	1803	27.87	1.29E-07	1.21E-05	no	protein binding	Tetratricopeptide repeat-containing domain; Tetratricopeptide-like helical domain superfamily; Tetratricopeptide repeat 1; Tetratricopeptide repeat
PMUR_0002000	Phyca11_508348, PITG_03940T0, KUF90639, Phal15119, PMUR_0002000, PVIT_0017186	183	27.65	1.45E-07	1.32E-05	no	catalytic activity; integral component of membrane; molybdenum ion binding; pyridoxal phosphate binding	Molybdenum cofactor sulfurase, C-terminal; MOSC, N-terminal beta barrel; Pyruvate kinase-like, insert domain superfamily
PMUR_0000736	Phyca11_503654, PITG_03576T0, KUF64923, Phal09687, PMUR_0000736, PVIT_0000762	642	27.44	1.62E-07	1.45E-05	no	transferase activity	tRNA methyltransferase TRMD/TRM10-type domain; tRNA (guanine-N1-)-methyltransferase, eukaryotic
PMUR_0009831	Phyca11_550767, PITG_12465T0, KUF64516, Phal03922, PMUR_0009831, PVIT_0018529	423	27.39	1.67E-07	1.45E-05	no	adenylate kinase activity; ATP binding; nucleus; ATPase activity; nucleotide phosphorylation	P-loop containing nucleoside triphosphate hydrolase
PMUR_0010613	Phyca11_504513, PITG_01923T0, KUF85018, Phal06254, PMUR_0010613, PVIT_0013712	1875	25.76	3.86E-07	3.30E-05	no	DNA binding; nucleus; transcription, DNA-templated	PWWP domain; Transcription elongation factor S-II, central domain
PMUR_0012785	Phyca11_111612, PITG_13262T0, KUF80949, Phal11076, PMUR_0012785, PVIT_0012692	1497	25.37	4.72E-07	3.95E-05	no	cystathionine beta-synthase activity; cysteine synthase activity; cytoplasm; cysteine biosynthetic process from serine; vesicle docking involved in exocytosis; integral component of membrane; vesicle-mediated transport; cysteine biosynthetic process via cystathionine; pyridoxal phosphate binding	Cysteine synthase/cystathionine beta-synthase, pyridoxal-phosphate attachment site; CBS domain; Pyridoxal-phosphate dependent enzyme; Sec1-like protein; Cystathionine beta-synthase
PMUR_0007052	Phyca11_548231, PITG_17272T0, KUF85373, Phal03255, PMUR_0007052, PVIT_0007149	1779	25.16	5.29E-07	4.33E-05	no	RNA binding; binding	Armadillo-like helical; Armadillo-type fold; Pumilio RNA-binding repeat
PMUR_0001201	Phyca11_556876, PITG_02622T0, KUF85772, Phal06972, PMUR_0001201, PVIT_0001162	1920	24.66	6.83E-07	5.49E-05	no	aspartic-type endopeptidase activity; intracellular; nucleus; proteolysis; zinc ion binding; response to light stimulus; regulation of flower development; membrane; protein catabolic process	B-box-type zinc finger; Aspartic peptidase A1 family; Zinc finger, RING/FYVE/PHD-type; Aspartic peptidase domain superfamily
PMUR_0008437	Phyca11_507649, PITG_02948T0, KUF66946, Phal10416, PMUR_0008437, PVIT_0001408	1029	23.97	9.79E-07	7.71E-05	no	ATP binding; protein folding; response to heat; heat shock protein binding; metal ion binding; unfolded protein binding	DnaJ domain, conserved site; Heat shock protein DnaJ, cysteine-rich domain; DnaJ domain; Chaperone DnaJ, C-terminal; Chaperone DnaJ; HSP40/DnaJ peptide-binding
PMUR_0004280	Phyca11_505827, PITG_15846T0, KUF80590, Phal00682, PMUR_0004280, PVIT_0003668	2298	23.41	1.31E-06	1.01E-04	no	membrane; integral component of membrane	Calcium-dependent channel, 7TM region, putative phosphate; 10TM putative phosphate transporter, cytosolic domain
PMUR_0005674	Phyca11_511802, PITG_23191T0, KUF75376, Phal10091, PMUR_0005674, PVIT_0019782	492	23.27	1.41E-06	1.07E-04	no	mRNA splicing, via spliceosome; RNA binding; protein binding; U1 snRNP; viral nucleocapsid; catalytic step 2 spliceosome	LSM domain, eukaryotic/archaea-type; Tetratricopeptide repeat-containing domain; LSM domain superfamily; Tetratricopeptide-like helical domain superfamily; Tetratricopeptide repeat
PMUR_0009083	Phyca11_120327, PITG_11108T0, KUF76033, Phal00418, PMUR_0009083, PVIT_0006615	324	22.83	1.77E-06	1.32E-04	yes		LSM domain, eukaryotic/archaea-type; LSM domain superfamily
PMUR_0003444	Phyca11_531591, PITG_02325T0, KUG00912, Phal04997, PMUR_0003444, PVIT_0000931	1236	22.64	1.95E-06	1.43E-04	yes	nucleotide binding; cation transport; membrane; integral component of membrane; hydrolase activity; cation-transporting ATPase activity; metal ion transport; metal ion binding; cation transmembrane transport	Heavy metal-associated domain, HMA

Tested gene	Orthogroup	Align. size	Delta LRT	*P* -value	*Q* -value	Secreted	GO terms	InterPro domains
PMUR_0002428	Phyca11_529777, PITG_16342T0, KUG00159, Phal05532, PMUR_0002428, PVIT_0011072	378	22.59	2.00E-06	1.43E-04	no	transcription coactivator activity; nucleus; mitochondrion; cytosol; regulation of transcription from RNA polymerase II promoter; membrane; integral component of membrane; glyoxalase III activity; methylglyoxal catabolic process to D-lactate via S-lactoyl-glutathione; lactate biosynthetic process; cellular response to hydrogen peroxide	DJ-1/PfpI
PMUR_0002711	Phyca11_5748, PITG_04644T0, KUG00479, Phal03556, PMUR_0002711, PVIT_0011528	1584	21.71	3.18E-06	2.24E-04	no	mRNA (guanine-N7-)-methyltransferase activity; protein binding; nucleus; mRNA cap binding complex; 7-methylguanosine mRNA capping; RNA (guanine-N7)-methylation	WW domain; mRNA (guanine-N(7))-methyltransferase domain; mRNA (guanine-N(7))-methyltransferase
PMUR_0000822	Phyca11_35790, PITG_07145T0, KUF77196, Phal04042, PMUR_0000822, PVIT_0005563	975	21.67	3.25E-06	2.24E-04	no	fatty acid biosynthetic process; malonyl-CoA decarboxylase activity	Malonyl-CoA decarboxylase, C-terminal
PMUR_0009071	Phyca11_509092, PITG_11127T0, KUF76536, Phal01636, PMUR_0009071, PVIT_0006602	1539	21.64	3.29E-06	2.24E-04	no	transport; retrograde vesicle-mediated transport, Golgi to ER; protein transport; COPI vesicle coat	Coatomer delta subunit; Longin-like domain superfamily
PMUR_0009216	Phyca11_546925, PITG_07262T0, KUF79909, Phal12526, PMUR_0009216, PVIT_0009773	2088	21.14	4.26E-06	2.85E-04	no		Ribosomal protein L30, conserved site; Ribosomal protein L30, N-terminal; Ribosomal protein L30, ferredoxin-like fold domain; FMP27, C-terminal; Ribosomal protein L7, eukaryotic
PMUR_0006586	Phyca11_526518, PITG_06171T0, KUF77017, Phal07554, PMUR_0006586, PVIT_0002687	3594	19.83	8.47E-06	5.57E-04	no	spliceosomal complex assembly; mRNA binding; binding; U2 snRNP; U12-type spliceosomal complex; U2-type prespliceosome; catalytic step 2 spliceosome	Splicing factor 3B subunit 1; Armadillo-like helical; Armadillo-type fold
PMUR_0008667	Phyca11_551967, PITG_15630T0, KUF94802, Phal09504, PMUR_0008667, PVIT_0019840	249	19.44	1.04E-05	6.74E-04	no	ESCRT II complex; structural molecule activity; protein homodimerization activity; protein targeting to vacuole involved in ubiquitin-dependent protein catabolic process via the multivesicular body sorting pathway	ArsR-like helix-turn-helix domain; ESCRT-II complex, Vps25 subunit; ESCRT-II complex, Vps25 subunit, N-terminal winged helix
PMUR_0008085	Phyca11_546359, PITG_13184T0, KUF81475, Phal04416, PMUR_0008085, PVIT_0016443	1263	19.20	1.18E-05	7.51E-04	no	membrane	Polycystin cation channel, PKD1/PKD2
PMUR_0002375	Phyca11_561297, PITG_11451T0, KUF91097, Phal05449, PMUR_0002375, PVIT_0005733	1938	18.80	1.45E-05	9.11E-04	no		
PMUR_0003716	Phyca11_530047, PITG_09305T0, KUG00458, Phal00064, PMUR_0003716, PVIT_0008141	585	18.72	1.51E-05	9.35E-04	no	recombinase activity; four-way junction DNA binding; DNA recombinase assembly; condensed nuclear chromosome; DNA binding; double-stranded DNA binding; single-stranded DNA binding; endodeoxyribonuclease activity; ATP binding; nucleoplasm; DNA repair; mitotic recombination; reciprocal meiotic recombination; DNA-dependent ATPase activity; response to ionizing radiation; strand invasion; chromosome organization involved in meiotic cell cycle	AAA+ ATPase domain; DNA recombination and repair protein Rad51-like, C-terminal; DNA recombination and repair protein RecA-like, ATP-binding domain; DNA recombination and repair protein, RecA-like; P-loop containing nucleoside triphosphate hydrolase
PMUR_0003497	Phyca11_528670, PITG_02447T0, KUF83950, Phal03756, PMUR_0003497, PVIT_0000984	666	18.69	1.54E-05	9.38E-04	no		NADH:ubiquinone oxidoreductase intermediate-associated protein 30; Galactose-binding-like domain superfamily
PMUR_0006334	Phyca11_110703, PITG_01431T0, KUF81519, Phal14129, PMUR_0006334, PVIT_0013064	1644	17.56	2.78E-05	1.66E-03	yes	carbohydrate metabolic process; beta-glucosidase activity; integral component of membrane; glycosyl compound metabolic process	Glycoside hydrolase family 1; Glycoside hydrolase superfamily
PMUR_0008841	Phyca11_511335, PITG_15139T0, KUF92685, Phal06804, PMUR_0008841, PVIT_0015324	2142	17.38	3.06E-05	1.81E-03	no	nucleotide binding; magnesium ion binding; phospholipid-translocating ATPase activity; ATP binding; plasma membrane; phospholipid transport; integral component of membrane; phospholipid translocation	P-type ATPase; P-type ATPase, subfamily IV; P-type ATPase, A domain superfamily; HAD superfamily; P-type ATPase, transmembrane domain superfamily; P-type ATPase, cytoplasmic domain N; P-type ATPase, phosphorylation site
PMUR_0004612	Phyca11_504053, PITG_14818T0, KUF90246, Phal13482, PMUR_0004612, PVIT_0019686	1851	17.34	3.13E-05	1.82E-03	no	GTPase activity; GTP binding	Guanylate-binding protein/Atlastin, C-terminal; Guanylate-binding protein, N-terminal; P-loop containing nucleoside triphosphate hydrolase
PMUR_0001580	Phyca11_545202, PITG_05541T0, KUF76734, Phal05708, PMUR_0001580, PVIT_0006114	933	17.21	3.35E-05	1.92E-03	no	endoplasmic reticulum membrane; protein N-linked glycosylation; oligosaccharide-lipid intermediate biosynthetic process; integral component of membrane; dolichyl pyrophosphate Man9GlcNAc2 alpha-1,3-glucosyltransferase activity	Glycosyl transferase, ALG6/ALG8
PMUR_0006626	Phyca11_19592, PITG_11966T0, KUF85566, Phal08576, PMUR_0006626, PVIT_0005945	1683	17.14	3.48E-05	1.97E-03	no	ATP binding; cytoplasm; protein refolding	Chaperonin Cpn60; Chaperonin Cpn60/TCP-1 family; GroEL-like apical domain superfamily; GroEL-like equatorial domain superfamily

Tested gene	Orthogroup	Align. size	Delta LRT	*P* -value	*Q* -value	Secreted	GO terms	InterPro domains
PMUR_0003710	Phyca11_510133, PITG_09345T0, KUF78834, Phal00069, PMUR_0003710, PVIT_0008133	588	16.69	4.41E-05	2.42E-03	no	ribosomal small subunit assembly; RNA binding; mRNA binding; structural constituent of ribosome; translation; small ribosomal subunit; rRNA binding; cytosolic small ribosomal subunit	Ribosomal protein S7, conserved site; Ribosomal protein S7 domain; Ribosomal protein S5/S7; Ribosomal protein S5/S7, eukaryotic/archaeal
PMUR_0005362	Phyca11_502585, PITG_14639T0, KUF79068, Phal06879, PMUR_0005362, PVIT_0016036	969	16.70	4.37E-05	2.42E-03	no	catalytic activity; transaminase activity; 4-hydroxy-tetrahydrodipicolinate reductase; biosynthetic process; lysine biosynthetic process via diaminopimelate; pyridoxal phosphate binding; oxidation-reduction process	Dihydrodipicolinate reductase, conserved site; Dihydrodipicolinate reductase, N-terminal; Aminotransferase, class I/classII; NAD(P)-binding domain; Dihydrodipicolinate reductase, C-terminal; Pyridoxal phosphate-dependent transferase, major domain; Pyridoxal phosphate-dependent transferase domain 1; Pyridoxal phosphate-dependent transferase
PMUR_0004615	Phyca11_560957, PITG_14806T0, KUF77657, Phal04782, PMUR_0004615, PVIT_0019688	591	16.54	4.77E-05	2.59E-03	no	TRAPP complex; Golgi vesicle transport	Transport protein particle (TRAPP) component; TRAPP I complex, subunit 5; NO signalling/Golgi transport ligand-binding domain superfamily
PMUR_0004268	Phyca11_505833, PITG_15831T0, KUF80602, Phal08044, PMUR_0004268, PVIT_0003655	534	16.46	4.98E-05	2.67E-03	no	ATP binding; mitochondrion; folic acid-containing compound biosynthetic process; 5-formyltetrahydrofolate cyclo-ligase activity; tetrahydrofolate interconversion; metal ion binding	5-formyltetrahydrofolate cyclo-ligase
PMUR_0007242	Phyca11_34812, PITG_09792T0, KUF80477, Phal07279, PMUR_0007242, PVIT_0004577	1200	16.37	5.20E-05	2.75E-03	no	nucleic acid binding; ATP-dependent RNA helicase activity; ATP binding; RNA secondary structure unwinding; ion binding	Helicase, C-terminal; DEAD/DEAH box helicase domain; Helicase superfamily 1/2, ATP-binding domain; RNA helicase, DEAD-box type, Q motif; P-loop containing nucleoside triphosphate hydrolase
PMUR_0006578	Phyca11_102887, PITG_19877T0, KUF79491, Phal10225, PMUR_0006578, PVIT_0018779	1110	16.17	5.80E-05	3.03E-03	no	GTPase activity; GTP binding	Pleckstrin homology domain; Guanylate-binding protein/Atlastin, C-terminal; Guanylate-binding protein, N-terminal; PH-like domain superfamily; P-loop containing nucleoside triphosphate hydrolase
PMUR_0003373	Phyca11_539851, PITG_03744T0, KUF64622, Phal02084, PMUR_0003373, PVIT_0009278	585	15.80	7.04E-05	3.63E-03	no	protein binding	Bromodomain, conserved site; Bromodomain; NET domain
PMUR_0006068	Phyca11_127589, PITG_06718T0, KUF78798, Phal13655, PMUR_0006068, PVIT_0008220	453	15.62	7.75E-05	3.94E-03	yes	cilium or flagellum-dependent cell motility; microtubule motor activity; axonemal dynein complex; microtubule-based movement; ATPase activity	EGF-like, conserved site; EGF-like domain; EGF-like domain, extracellular
PMUR_0000412	Phyca11_121892, PITG_09835T0, KUF84368, Phal00319, PMUR_0000412, PVIT_0010555	1044	15.35	8.91E-05	4.47E-03	no	protein binding; cytosol; COP9 signalosome; NEDD8-specific protease activity	Proteasome component (PCI) domain; ArsR-like helix-turn-helix domain
PMUR_0002201	Phyca11_538823, PITG_03169T0, KUF83342, Phal12579, PMUR_0002201, PVIT_0000369	765	15.29	9.20E-05	4.51E-03	no	metal ion binding	GPN-loop GTPase; P-loop containing nucleoside triphosphate hydrolase
PMUR_0005390	Phyca11_557901, PITG_14602T0, KUF84861, Phal04182, PMUR_0005390, PVIT_0016004	2628	15.30	9.19E-05	4.51E-03	no		
PMUR_0000189	Phyca11_6928, PITG_15920T0, KUF84589, Phal09845, PMUR_0000189, PVIT_0003723	693	15.08	1.03E-04	4.99E-03	no	integral component of membrane	Transmembrane protein TMEM64
PMUR_0011101	Phyca11_15660, PITG_10849T0, KUF80138, Phal11409, PMUR_0011101, PVIT_0017034	1290	14.95	1.10E-04	5.27E-03	no		
PMUR_0011413	Phyca11_502634, PITG_12837T0, KUF82068, Phal07425, PMUR_0011413, PVIT_0022458	762	14.93	1.12E-04	5.27E-03	no	nitrogen compound metabolic process; hydrolase activity, acting on carbon-nitrogen (but not peptide) bonds	Carbon-nitrogen hydrolase; MORN motif
PMUR_0004491	Phyca11_511651, PITG_12428T0, KUF91253, Phal00893, PMUR_0004491, PVIT_0018586	282	14.85	1.17E-04	5.45E-03	no	nucleus; nucleosome assembly; CAF-1 complex	Chromatin assembly factor 1 subunit A; Armadillo-like helical
PMUR_0005574	Phyca11_531963, PITG_06986T0, KUF97067, Phal10488, PMUR_0005574, PVIT_0018084	2154	14.82	1.18E-04	5.45E-03	no	nucleic acid binding; exonuclease activity; 5'-3' exoribonuclease activity; nucleus; nucleobase-containing compound metabolic process; RNA phosphodiester bond hydrolysis, exonucleolytic	Putative 5-3 exonuclease; 5'-3' exoribonuclease type 2; 5'-3' exoribonuclease
PMUR_0011524	Phyca11_506258, PITG_12916T0, KUF84984, Phal06271, PMUR_0011524, PVIT_0006426	1053	14.75	1.23E-04	5.60E-03	no	cysteine-type endopeptidase activity; extracellular space; lysosome; proteolysis; cysteine-type peptidase activity; membrane; proteolysis involved in cellular protein catabolic process	Cysteine peptidase, cysteine active site; Cysteine peptidase, histidine active site; Cysteine peptidase, asparagine active site; Peptidase C1A, papain C-terminal; Peptidase C1A

Tested gene	Orthogroup	Align. size	Delta LRT	*P* -value	*Q* -value	Secreted	GO terms	InterPro domains
PMUR_0011039	Phyca11_511466, PITG_21071T0, KUF97922, Phal11133, PMUR_0011039, PVIT_0009159	2070	14.69	1.27E-04	5.73E-03	no	nucleotide binding; RNA binding; aminoacyl-tRNA ligase activity; threonine-tRNA ligase activity; ATP binding; cytoplasm; tRNA aminoacylation for protein translation; threonyl-tRNA aminoacylation; tRNA aminoacylation	Aminoacyl-tRNA synthetase, class II (G/ P/ S/T); TGS; Anticodon-binding; Aminoacyl-tRNA synthetase, class II; Threonyl/alanyl tRNA synthetase, SAD; Threonine-tRNA ligase, class IIa; Beta-grasp domain superfamily; TGS-like; Threonyl/alanyl tRNA synthetase, class II-like, putative editing domain superfamily
PMUR_0010795	Phyca11_552406, PITG_20520T0, KUF74545, Phal11007, PMUR_0010795, PVIT_0008057	1191	14.03	1.80E-04	8.03E-03	no	hydrolase activity	UbiB domain; Protein kinase-like domain superfamily
PMUR_0002374	Phyca11_504193, PITG_11457T0, KUF91107, Phal05450, PMUR_0002374, PVIT_0005734	3966	13.70	2.15E-04	9.39E-03	no	phosphoribosylformylglycinamidine synthase activity; ATP binding; cytoplasm; 'de novo' IMP biosynthetic process; glutamine metabolic process	PurM-like, C-terminal domain; PurM-like, N-terminal domain; Glutamine amidotransferase; Phosphoribosylformylglycinamidine synthase
PMUR_0007961	Phyca11_542166, PITG_02035T0, KUG00299, Phal08830, PMUR_0007961, PVIT_0007421	951	13.70	2.14E-04	9.39E-03	no	protein binding	WD40-repeat-containing domain; Six-bladed beta-propeller, TolB-like; WD40/YVTN repeat-like-containing domain superfamily; WD40 repeat
PMUR_0003742	Phyca11_511439, PITG_09282T0, KUG00452, Phal10033, PMUR_0003742, PVIT_0008172	3102	13.65	2.20E-04	9.50E-03	no	nucleotide binding; ATP binding; endoplasmic reticulum; integral component of plasma membrane; integral component of membrane; cation-transporting ATPase activity; metal ion binding; cation transmembrane transport	Cation-transporting P-type ATPase, N-terminal; Cation-transporting P-type ATPase, C-terminal; P-type ATPase; P-type ATPase, A domain superfamily; HAD superfamily; P-type ATPase, transmembrane domain superfamily; P-type ATPase, cytoplasmic domain N; P-type ATPase, phosphorylation site
PMUR_0005881	Phyca11_125088, PITG_17784T0, KUF78421, Phal06976, PMUR_0005881, PVIT_0003622	855	13.52	2.36E-04	1.00E-02	no	methyltransferase activity; methylation	SAM-dependent methyltransferase RsmB/NOP2-type; RNA (C5-cytosine) methyltransferase
PMUR_0005965	Phyca11_530811, PITG_17370T0, KUF77762, Phal03380, PMUR_0005965, PVIT_0007077	1599	13.51	2.37E-04	1.00E-02	no	vacuole inheritance; cytosol; Golgi to endosome transport; endocytosis; cytoplasmic side of endosome membrane; Rab GTPase binding; endosomal vesicle fusion; metal ion binding	FYVE zinc finger; Zinc finger, FYVE-related; Zinc finger, FYVE/PHD-type; Zinc finger, RING/FYVE/PHD-type; START-like domain superfamily
PMUR_0005763	Phyca11_534052, PITG_21886T0, KUF76579, Phal04857, PMUR_0005763, PVIT_0012793	2190	12.87	3.34E-04	1.40E-02	no	catalytic activity; histidine-tRNA ligase activity; ATP binding; cytoplasm; mitochondrion; histidyl-tRNA aminoacylation; mitochondrial translation	Anticodon-binding; Aminoacyl-tRNA synthetase, class II; Aromatic amino acid lyase; Histidine-tRNA ligase/ATP phosphoribosyltransferase regulatory subunit; Histidine-tRNA ligase; L-Aspartase-like
PMUR_0001138	Phyca11_545851, PITG_01412T0, KUF77091, Phal03341, PMUR_0001138, PVIT_0013050	276	12.83	3.40E-04	1.41E-02	no	ion transport; ion transmembrane transporter activity; membrane; integral component of membrane; ion transmembrane transport; transmembrane transport	Tricarboxylate/iron carrier
PMUR_0001623	Phyca11_527238, PITG_05482T0, KUF80011, Phal09010, PMUR_0001623, PVIT_0006066	654	12.51	4.05E-04	1.66E-02	no		PUA-like superfamily
PMUR_0005464	Phyca11_511142, PITG_21377T0, KUF82285, Phal13589, PMUR_0005464, PVIT_0011409	579	12.39	4.32E-04	1.75E-02	no	calcium ion binding	EF-Hand 1, calcium-binding site; EF-hand domain; EF-hand domain pair
PMUR_0001936	Phyca11_509604, PITG_00973T0, KUF90323, Phal05387, PMUR_0001936, PVIT_0007287	600	12.24	4.69E-04	1.88E-02	no		von Willebrand factor, type A; Copine
PMUR_0006766	Phyca11_558170, PITG_13014T0, KUF76541, Phal03980, PMUR_0006766, PVIT_0021050	1161	12.06	5.14E-04	2.05E-02	no	DNA binding; proteolysis; metallopeptidase activity	Peptidase M24; ArsR-like helix-turn-helix domain; PA2G4 family
PMUR_0011519	Phyca11_110421, PITG_12919T0, KUF84986, Phal06266, PMUR_0011519, PVIT_0006431	3036	12.00	5.32E-04	2.10E-02	no		
PMUR_0008412	Phyca11_543837, PITG_18633T0, KUF82234, Phal13120, PMUR_0008412, PVIT_0020500	921	11.94	5.49E-04	2.14E-02	no	pseudouridine synthesis; RNA binding; RNA processing; tRNA modification; RNA modification; pseudouridine synthase activity; mRNA pseudouridine synthesis	PUA domain; Pseudouridine synthase II, N-terminal; tRNA pseudouridine synthase II, TruB; Pseudouridine synthase, catalytic domain superfamily
PMUR_0006895	Phyca11_504209, PITG_11425T0, KUF82386, Phal13957, PMUR_0006895, PVIT_0009011	1974	11.53	6.83E-04	2.61E-02	no	telomere maintenance via recombination; telomerase activity; protein binding; telomerase holoenzyme complex; RNA-dependent DNA biosynthetic process; nuclear matrix	WD40 repeat, conserved site; Transcriptional repressor Tup1, N-terminal; WD40-repeat-containing domain; Six-bladed beta-propeller, TolB-like; WD40 repeat; G-protein beta WD-40 repeat
PMUR_0007560	Phyca11_503483, PITG_03811T0, KUF85587, Phal06083, PMUR_0007560, PVIT_0009341	768	11.54	6.80E-04	2.61E-02	no	kinase activity; phosphorylation	HAD superfamily
PMUR_0001748	Phyca11_571032, PITG_04715T0, KUF80265, Phal13768, PMUR_0001748, PVIT_0015920	960	11.34	7.57E-04	2.87E-02	no	pantothenate kinase activity; ATP binding; coenzyme A biosynthetic process; integral component of membrane; phosphorylation	Type II pantothenate kinase
PMUR_0009594	Phyca11_508289, PITG_17093T0, KUF79517, Phal06874, PMUR_0009594, PVIT_0010310	453	11.33	7.65E-04	2.87E-02	no	ribosomal large subunit assembly; structural constituent of ribosome; translation; cytosolic large ribosomal subunit; assembly of large subunit precursor of preribosome	Ribosomal protein L24e, conserved site; Ribosomal protein L24e-related

Tested gene	Orthogroup	Align. size	Delta LRT	*P* -value	*Q* -value	Secreted	GO terms	InterPro domains
PMUR_0005817	Phyca11_106683, PITG_10252T0, KUG00411, Phal14188, PMUR_0005817, PVIT_0003069	414	11.16	8.34E-04	3.10E-02	no		
PMUR_0005400	Phyca11_36571, PITG_14593T0, KUF76064, Phal13378, PMUR_0005400, PVIT_0015994	1278	11.11	8.60E-04	3.17E-02	no	binding; protein binding	Armadillo-like helical; Armadillo-type fold; Armadillo
PMUR_0009691	Phyca11_509394, PITG_16466T0, KUF74988, Phal11912, PMUR_0009691, PVIT_0013247	624	11.06	8.84E-04	3.20E-02	no		
PMUR_0011370	Phyca11_5374, PITG_14971T0, KUF76285, Phal04784, PMUR_0011370, PVIT_0004681	2307	11.06	8.82E-04	3.20E-02	no	protein kinase activity; ATP binding; protein phosphorylation; membrane; kinase activity; phosphorylation	Protein kinase domain; Protein kinase-like domain superfamily
PMUR_0006889	Phyca11_571513, PITG_11565T0, KUF75992, Phal06787, PMUR_0006889, PVIT_0009006	444	10.89	9.69E-04	3.48E-02	no	RNA ligase (ATP) activity; ATP binding; tRNA splicing, via endonucleolytic cleavage and ligation	NEDD4-binding protein 2-like 2; P-loop containing nucleoside triphosphate hydrolase
PMUR_0006042	Phyca11_5314, PITG_09682T0, KUF79157, Phal00585, PMUR_0006042, PVIT_0004519	606	10.84	9.92E-04	3.50E-02	no		
PMUR_0012189	Phyca11_534810, PITG_00845T0, KUF77237, Phal09887, PMUR_0012189, PVIT_0007185	1404	10.84	9.93E-04	3.50E-02	no	3',5'-cyclic-nucleotide phosphodiesterase activity; protein binding; signal transduction; phosphoric diester hydrolase activity; metal ion binding	3'5'-cyclic nucleotide phosphodiesterase, conserved site; 3'5'-cyclic nucleotide phosphodiesterase, catalytic domain; GAF domain; HD/PDEase domain
PMUR_0001089	Phyca11_7431, PITG_01363T0, KUF97689, Phal01606, PMUR_0001089, PVIT_0013001	1236	10.82	1.01E-03	3.52E-02	no		
PMUR_0008270	Phyca11_99088, PITG_06848T0, KUF86793, Phal09162, PMUR_0008270, PVIT_0008522	705	10.60	1.13E-03	3.88E-02	no	RNA methylation; tRNA nucleoside ribose methylation; cytoplasmic translation; cytoplasm; methyltransferase activity; tRNA methyltransferase activity; methylation	Ribosomal RNA methyltransferase FtsJ domain; Ribosomal RNA large subunit methyltransferase E
PMUR_0008538	Phyca11_11261, PITG_00531T0, KUF95640, Phal08382, PMUR_0008538, PVIT_0001747	1272	10.61	1.12E-03	3.88E-02	no	protein-lysine N-methyltransferase activity; peptidyl-lysine monomethylation	Rubisco LSMT, substrate-binding domain
PMUR_0001052	Phyca11_7451, PITG_01313T0, KUF83241, Phal03492, PMUR_0001052, PVIT_0012961	975	10.58	1.14E-03	3.89E-02	no	peroxisomal membrane; peroxisome organization	Peroxisome membrane protein, Pex16
PMUR_0004784	Phyca11_568079, PITG_03343T0, KUF76147, Phal06948, PMUR_0004784, PVIT_0000531	891	10.50	1.20E-03	4.03E-02	no		
PMUR_0003899	Phyca11_118786, PITG_00620T0, KUF76974, Phal03586, PMUR_0003899, PVIT_0001830	888	10.43	1.24E-03	4.16E-02	no	nucleotide binding; microtubule motor activity; ATP binding; kinesin complex; microtubule; microtubule-based movement; microtubule binding; ATPase activity	Kinesin motor domain; P-loop containing nucleoside triphosphate hydrolase
PMUR_0005922	Phyca11_4436, PITG_04425T0, KUF76438, Phal11717, PMUR_0005922, PVIT_0016636	588	10.36	1.29E-03	4.27E-02	no	protein polyubiquitination; protein binding; ATP binding; nucleus; cytoplasm; ubiquitin protein ligase binding; proteasome-mediated ubiquitin-dependent protein catabolic process; ubiquitin protein ligase activity	Ubiquitin-conjugating enzyme, active site; Ubiquitin-conjugating enzyme E2; Ubiquitin-associated domain; UBA-like superfamily; Ubiquitin-conjugating enzyme/RWD-like
PMUR_0009868	Phyca11_506559, PITG_13346T0, KUG01598, Phal06319, PMUR_0009868, PVIT_0012642	1173	10.28	1.35E-03	4.43E-02	no	protein binding	IQ motif, EF-hand binding site
PMUR_0010618	Phyca11_106461, PITG_16210T0, KUF76884, Phal13557, PMUR_0010618, PVIT_0013314	810	10.10	1.48E-03	4.84E-02	no	catalytic activity; ATP binding; cellular protein modification process; ligase activity	Tubulin-tyrosine ligase/Tubulin polyglutamylase; Tubulin--tyrosine ligase-like protein 12; ATP-grasp fold, subdomain 1
PMUR_0000724	Phyca11_540044, PITG_03594T0, KUF64885, Phal09375, PMUR_0000724, PVIT_0000771	2784	9.95	1.61E-03	5.17E-02	no	protein binding	WD40 repeat, conserved site; WD40-repeat-containing domain; WD40/YVTN repeat-like-containing domain superfamily; WD40 repeat
PMUR_0002151	Phyca11_10446, PITG_01043T0, KUF76949, Phal04939, PMUR_0002151, PVIT_0009513	1452	9.94	1.62E-03	5.17E-02	no	protein kinase activity; protein serine/threonine kinase activity; ATP binding; intracellular; protein phosphorylation; peptidyl-serine phosphorylation; intracellular signal transduction	Serine/threonine-protein kinase, active site; Protein kinase, ATP binding site; Protein kinase domain; Protein kinase-like domain superfamily; PH-like domain superfamily
PMUR_0013320	Phyca11_108908, PITG_15417T0, KUG00710, Phal10170, PMUR_0013320, PVIT_0018693	720	9.93	1.62E-03	5.17E-02	no	asparagine synthase (glutamine-hydrolyzing) activity; cytosol; asparagine biosynthetic process; glutamine metabolic process; protein homodimerization activity	Asparagine synthase; Glutamine amidotransferase type 2 domain; Rossmann-like alpha/beta/alpha sandwich fold
PMUR_0005360	Phyca11_557962, PITG_15641T0, KUG00944, Phal07048, PMUR_0005360, PVIT_0016038	777	9.85	1.70E-03	5.37E-02	no		
PMUR_0002730	Phyca11_83303, PITG_04609T0, KUF79936, Phal02515, PMUR_0002730, PVIT_0011507	225	9.79	1.75E-03	5.49E-02	no		
PMUR_0003509	Phyca11_502845, PITG_19128T0, KUF77603, Phal09915, PMUR_0003509, PVIT_0020040	1257	9.74	1.80E-03	5.62E-02	no		DnaJ domain, conserved site; DnaJ domain
PMUR_0005937	Phyca11_558581, PITG_04452T0, KUF79007, Phal04815, PMUR_0005937, PVIT_0016653	1212	9.63	1.92E-03	5.93E-02	no	ATP binding; ATP synthesis coupled proton transport; proton-transporting ATP synthase complex, catalytic core F(1); proton-transporting ATP synthase activity, rotational mechanism; proton-transporting ATPase activity, rotational mechanism	ATPase, F1/V1/A1 complex, alpha/beta subunit, nucleotide-binding domain; P-loop containing nucleoside triphosphate hydrolase

Tested gene	Orthogroup	Align. size	Delta LRT	*P* -value	*Q* -value	Secreted	GO terms	InterPro domains
PMUR_0007380	Phyca11_528591, PITG_18185T0, KUF87125, Phal07119, PMUR_0007380, PVIT_0019958	1413	9.46	2.10E-03	6.43E-02	no	catalytic activity	Phospholipase D/Transphosphatidylase; Phospholipase D-like domain
PMUR_0004545	Phyca11_128667, PITG_18992T0, KUF90381, Phal01553, PMUR_0004545, PVIT_0018936	1221	9.42	2.14E-03	6.52E-02	no	integral component of membrane; transferase activity, transferring acyl groups other than amino-acyl groups	Acyltransferase 3
PMUR_0003495	Phyca11_549442, PITG_02445T0, KUF83940, Phal03758, PMUR_0003495, PVIT_0000982	651	9.40	2.17E-03	6.54E-02	no		
PMUR_0005540	Phyca11_531975, PITG_07024T0, KUG02032, Phal09272, PMUR_0005540, PVIT_0018047	1521	9.35	2.23E-03	6.69E-02	no	nucleotide binding; catalytic activity; IMP dehydrogenase activity; cytoplasm; purine nucleotide biosynthetic process; GMP biosynthetic process; oxidoreductase activity; metal ion binding; oxidation-reduction process	IMP dehydrogenase / GMP reductase, conserved site; CBS domain; IMP dehydrogenase/GMP reductase; Inosine-5'-monophosphate dehydrogenase; Aldolase-type TIM barrel
PMUR_0007952	Phyca11_100998, PITG_14934T0, KUF76276, Phal12359, PMUR_0007952, PVIT_0004686	1170	9.26	2.34E-03	6.98E-02	no		
PMUR_0011522	Phyca11_533911, PITG_12914T0, KUF84990, Phal06269, PMUR_0011522, PVIT_0006427	1797	9.21	2.41E-03	7.11E-02	no	signal transduction; single-organism cellular process; phosphatidylinositol dephosphorylation	Rho GTPase-activating protein domain; Inositol polyphosphate-related phosphatase; Endonuclease/exonuclease/phosphatase; Rho GTPase activation protein; Immunoglobulin-like fold
PMUR_0000424	Phyca11_121943, PITG_08640T0, KUF97780, Phal01840, PMUR_0000424, PVIT_0010567	927	9.16	2.47E-03	7.25E-02	no	1-phosphatidylinositol 4-kinase activity; intracellular; plasma membrane; kinase activity; phosphatidylinositol phosphorylation; metal ion binding; phosphatidylinositol-mediated signaling	Phosphatidylinositol 3/4-kinase, conserved site; Phosphatidylinositol 3-/4-kinase, catalytic domain; Pleckstrin homology domain; Phosphatidylinositol kinase; Protein kinase-like domain superfamily; Zinc finger, FYVE/PHD-type; PH-like domain superfamily; Zinc finger, RING/FYVE/PHD-type
PMUR_0000516	Phyca11_534969, PITG_00361T0, KUF77127, Phal02770, PMUR_0000516, PVIT_0001618	840	9.13	2.52E-03	7.32E-02	no		YAP-binding/ALF4/Glomulin; Glomulin/ALF4
PMUR_0005398	Phyca11_4109, PITG_14595T0, KUF76069, Phal13376, PMUR_0005398, PVIT_0015996	414	9.12	2.53E-03	7.32E-02	no		
PMUR_0004715	Phyca11_104632, PITG_00025T0, KUF92809, Phal04027, PMUR_0004715, PVIT_0006794	1446	9.06	2.61E-03	7.48E-02	no	membrane; metal ion binding	Zinc finger, RING-type, conserved site; Zinc finger, RING-type; Zinc finger, RING/FYVE/PHD-type
PMUR_0001364	Phyca11_526284, PITG_10160T0, KUF83843, Phal11516, PMUR_0001364, PVIT_0007458	1896	9.04	2.64E-03	7.53E-02	yes	NADPH-hemoprotein reductase activity; endoplasmic reticulum membrane; FMN binding; integral component of membrane; oxidoreductase activity; [methionine synthase] reductase activity; oxidation-reduction process	Oxidoreductase FAD/NAD(P)-binding; Flavoprotein pyridine nucleotide cytochrome reductase; FAD-binding, type 1; Flavodoxin/nitric oxide synthase; Ferredoxin reductase-type FAD-binding domain; Flavodoxin-like; Riboflavin synthase-like beta-barrel
PMUR_0002175	Phyca11_558908, PITG_03094T0, KUF81709, Phal01416, PMUR_0002175, PVIT_0000339	441	9.00	2.70E-03	7.58E-02	no	integral component of membrane	
PMUR_0011785	Phyca11_566753, PITG_01674T0, KUG00358, Phal10720, PMUR_0011785, PVIT_0005446	900	9.01	2.69E-03	7.58E-02	no		P-loop containing nucleoside triphosphate hydrolase
PMUR_0003036	Phyca11_131317, PITG_20288T0, KUF95286, Phal10374, PMUR_0003036, PVIT_0012240	777	8.94	2.80E-03	7.80E-02	yes		
PMUR_0006956	Phyca11_507023, PITG_04970T0, KUF86944, Phal08323, PMUR_0006956, PVIT_0016512	2853	8.84	2.94E-03	8.15E-02	no		
PMUR_0010865	Phyca11_511575, PITG_12694T0, KUF81275, Phal00713, PMUR_0010865, PVIT_0013480	873	8.83	2.97E-03	8.16E-02	no	1-alkyl-2-acetylglycerophosphocholine esterase activity; protein binding; intracellular; vacuolar transport; hydrolase activity	WD40-repeat-containing domain; Gamma-secretase aspartyl protease complex, presenilin enhancer-2 subunit; WD40/YVTN repeat-like-containing domain superfamily; WD40 repeat; G-protein beta WD-40 repeat
PMUR_0008878	Phyca11_506576, PITG_15467T0, KUF80863, Phal10188, PMUR_0008878, PVIT_0020102	720	8.75	3.10E-03	8.48E-02	no		
PMUR_0010656	Phyca11_551908, PITG_15552T0, KUF81933, Phal02609, PMUR_0010656, PVIT_0021188	1110	8.73	3.13E-03	8.50E-02	no	phosphatidylinositol phosphate kinase activity; phosphatidylinositol metabolic process; phosphatidylinositol phosphorylation	Phosphatidylinositol-4-phosphate 5-kinase, core; Phosphatidylinositol-4-phosphate 5-kinase; Phosphatidylinositol-4-phosphate 5-kinase, C-terminal; Phosphatidylinositol-4-phosphate 5-kinase, N-terminal
PMUR_0006542	Phyca11_539182, PITG_06792T0, KUF86444, Phal07324, PMUR_0006542, PVIT_0008465	2760	8.63	3.30E-03	8.90E-02	no	nucleic acid binding; DNA binding; ATP-dependent DNA helicase activity; ATP binding; nucleus; nucleobase-containing compound metabolic process; ATP-dependent helicase activity; zinc ion binding; DNA duplex unwinding	DNA/RNA helicase, ATP-dependent, DEAH-box type, conserved site; Zinc finger, RING-type; Helicase-like, DEXD box c2 type; ATP-dependent helicase, C-terminal; DEAD2; Helicase superfamily 1/2, ATP-binding domain, DinG/Rad3-type; ATP-dependent helicase Rad3/Chl1-like; Zinc finger, RING/FYVE/PHD-type; P-loop containing nucleoside triphosphate hydrolase

Tested gene	Orthogroup	Align. size	Delta LRT	*P* -value	*Q* -value	Secreted	GO terms	InterPro domains
PMUR_0009488	Phyca11_11491, PITG_16813T0, KUF96155, Phal10207, PMUR_0009488, PVIT_0022045	1395	8.59	3.38E-03	9.04E-02	no	protein serine/threonine kinase activity; ATP binding; protein phosphorylation	RIO kinase, conserved site; RIO kinase; Serine/threonine-protein kinase Rio1; Protein kinase-like domain superfamily
PMUR_0011335	Phyca11_538054, PITG_12876T0, KUF84254, Phal07415, PMUR_0011335, PVIT_0022472	471	8.55	3.45E-03	9.18E-02	no	nucleotide binding; nucleic acid binding	RNA recognition motif domain
PMUR_0005097	Phyca11_18948, PITG_17384T0, KUF87775, Phal09280, PMUR_0005097, PVIT_0019364	876	8.53	3.49E-03	9.23E-02	no	protein binding	Tetratricopeptide repeat-containing domain; Tetratricopeptide-like helical domain superfamily; Tetratricopeptide repeat
PMUR_0009825	Phyca11_550750, PITG_12479T0, KUF64498, Phal02835, PMUR_0009825, PVIT_0018522	399	8.48	3.59E-03	9.43E-02	no		
PMUR_0004175	Phyca11_41439, PITG_11813T0, KUF76193, Phal08159, PMUR_0004175, PVIT_0015139	462	8.44	3.67E-03	9.57E-02	no	mitochondrion; mitochondrial transport; nervous system development; kinesin binding	KIF-1 binding protein
PMUR_0011035	Phyca11_529546, PITG_21222T0, KUF97925, Phal11965, PMUR_0011035, PVIT_0009155	2226	8.39	3.77E-03	9.78E-02	no	intracellular protein transport; retrograde vesicle-mediated transport, Golgi to ER; Golgi organization; Golgi transport complex	Conserved oligomeric Golgi complex subunit 7
PMUR_0007898	Phyca11_509876, PITG_05224T0, KUG01522, Phal04316, PMUR_0007898, PVIT_0001977	423	8.33	3.91E-03	1.00E-01	no	microtubule motor activity; catalytic activity; 4-aminobutyrate transaminase activity; protein binding; ATP binding; kinesin complex; microtubule-based movement; microtubule binding; transaminase activity; gamma-aminobutyric acid metabolic process; ATPase activity; pyridoxal phosphate binding; phosphatidylinositol dephosphorylation	C2 domain; Inositol polyphosphate-related phosphatase; Kinesin motor domain; Endonuclease/exonuclease/phosphatase; 4-aminobutyrate aminotransferase, eukaryotic; Aminotransferase class-III; SMAD/FHA domain superfamily; Pyridoxal phosphate-dependent transferase, major domain; Pyridoxal phosphate-dependent transferase domain 1; Pyridoxal phosphate-dependent transferase; P-loop containing nucleoside triphosphate hydrolase
PMUR_0011824	Phyca11_121654, PITG_15623T0, KUF94800, Phal09498, PMUR_0011824, PVIT_0009166	1629	8.34	3.89E-03	1.00E-01	no	mRNA splicing, via spliceosome; RNA binding; binding; protein binding; U2-type catalytic step 1 spliceosome; catalytic step 2 spliceosome	MIF4G-like, type 3; Initiation factor eIF-4 gamma, MA3; Armadillo-type fold

Orthogroup: orthology group used for the branch-site test. Align. size: size of the alignment for the computation of likelihoods. Delta LRT: test statistic value for the likelihood ratio test. *P* -values were computed considering that the test statistic followed a chi-square distribution with 1 degree of freedom. *Q* -values were computed using a FDR procedure.

REFERENCES

Adhikari BN, Hamilton JP, Zerillo MM *et al.* (2013) Comparative genomics reveals insight into virulence strategies of plant pathogenic Oomycetes. *PLoS ONE*, **8**, e75072.

Bankevich A, Nurk S, Antipov D *et al.* (2012) SPAdes: a new genome assembly algorithm and its applications to single-cell sequencing. *Journal of Computational Biology*, **19**, 455–77.

Baxter L, Tripathy S, Ishaque N *et al.* (2010) Signatures of adaptation to obligate biotrophy in the *Hyaloperonospora arabidopsidis* genome. *Science*, **330**, 1549–1551.

Berlin K, Koren S, Chin C-S *et al.* (2015) Assembling large genomes with single-molecule sequencing and locality-sensitive hashing. *Nature Biotechnology*, **33**, 623–630.

Brilli M, Asquini E, Moser M *et al.* (2018) A multi-omics study of the grapevine-downy mildew (*Plasmopara viticola*) pathosystem unveils a complex protein coding- and noncoding-based arms race during infection. *Scientific Reports*, **8**, 757.

Chin C-S, Peluso P, Sedlazeck FJ *et al.* (2016) Phased diploid genome assembly with single-molecule real-time sequencing. *Nature Methods*, **13**, 1050–1054.

Cock PJA, Grüning BA, Paszkiewicz K, Pritchard L (2013) Galaxy tools and workflows for sequence analysis with applications in molecular plant pathology. *PeerJ*, **1**, e167.

Derevnina L, Chin-Wo-Reyes S, Martin F *et al.* (2015) Genome sequence and architecture of the tobacco downy mildew pathogen *Peronospora tabacina*. *Molecular Plant-Microbe Interactions*, **28**, 1198–1215.

Dobin A, Davis CA, Schlesinger F *et al.* (2013) STAR: ultrafast universal RNA-seq aligner. *Bioinformatics*, **29**, 15–21.

Dussert Y, Gouzy J, Richart-Cervera S *et al.* (2016) Draft genome sequence of *Plasmopara viticola*, the grapevine downy mildew pathogen. *Genome Announcements*, **4**, e00987-16.

Grabherr MG, Haas BJ, Yassour M *et al.* (2011) Full-length transcriptome assembly from RNA-Seq data without a reference genome. *Nature Biotechnology*, **29**, 644–652.

Haas BJ, Kamoun S, Zody MC *et al.* (2009) Genome sequence and analysis of the Irish potato famine pathogen *Phytophthora infestans*. *Nature*, **461**, 393–398.

Hoff KJ, Lange S, Lomsadze A, Borodovsky M, Stanke M (2016) BRAKER1: Unsupervised RNA-Seq-Based Genome Annotation with GeneMark-ET and AUGUSTUS. *Bioinformatics*, **32**, 767–769.

Holt C, Yandell M (2011) MAKER2: an annotation pipeline and genome-database management tool for second-generation genome projects. *BMC Bioinformatics*, **12**, 491.

Jiang RHY, de Bruijn I, Haas BJ *et al.* (2013) Distinctive expansion of potential virulence genes in the genome of the Oomycete fish pathogen *Saprolegnia parasitica*. *PLoS Genetics*, **9**, e1003272.

Kemen E, Gardiner A, Schultz-Larsen T *et al.* (2011) Gene gain and loss during evolution of obligate parasitism in the white rust pathogen of *Arabidopsis thaliana*. *PLoS Biology*, **9**, e1001094.

Korf I (2004) Gene finding in novel genomes. *BMC Bioinformatics*, **5**, 59.

Lamour KH, Mudge J, Gobena D *et al.* (2012) Genome sequencing and mapping reveal loss of heterozygosity as a mechanism for rapid adaptation in the vegetable pathogen *Phytophthora capsici*. *Molecular Plant-Microbe Interactions*, **25**, 1350–1360.

Lévesque CA, Brouwer H, Cano L *et al.* (2010) Genome sequence of the necrotrophic plant pathogen *Pythium ultimum* reveals original pathogenicity mechanisms and effector repertoire. *Genome Biology*, **11**, R73.

Li H, Durbin R (2009) Fast and accurate short read alignment with Burrows-Wheeler transform. *Bioinformatics*, **25**, 1754–1760.

Liu H, Ma X, Yu H *et al.* (2016) Genomes and virulence difference between two physiological races of *Phytophthora nicotianae*. *GigaScience*, **5**, 3.

McMullan M, Gardiner A, Bailey K *et al.* (2015) Evidence for suppression of immunity as a driver for genomic introgressions and host range expansion in races of *Albugo candida*, a generalist parasite. *eLife*, **4**, e04550.

Sharma R, Xia X, Cano LM *et al.* (2015) Genome analyses of the sunflower pathogen *Plasmopara halstedii* provide insights into effector evolution in downy mildews and *Phytophthora*. *BMC Genomics*, **16**, 741.

Simão FA, Waterhouse RM, Ioannidis P, Kriventseva E V., Zdobnov EM (2015) BUSCO: assessing genome assembly and annotation completeness with single-copy orthologs. *Bioinformatics*, **31**, 3210–3212.

Tyler BM, Tripathy S, Zhang X *et al.* (2006) *Phytophthora* genome sequences uncover evolutionary origins and mechanisms of pathogenesis. *Science*, **313**, 1261–1266.

Wicker T, Sabot F, Hua-Van A *et al.* (2007) A unified classification system for eukaryotic transposable elements. *Nature Reviews Genetics*, **8**, 973–982.

Yin L, An Y, Qu J *et al.* (2017) Genome sequence of *Plasmopara viticola* and insight into the pathogenic mechanism. *Scientific Reports*, **7**, 46553.
